# Advances in immunology and immunotherapy for mesenchymal gastrointestinal cancers

**DOI:** 10.1186/s12943-023-01770-6

**Published:** 2023-04-18

**Authors:** Bo Li, Hui Chen, Shaohua Yang, Feng Chen, Liangliang Xu, Yan Li, Mingzhe Li, Chengming Zhu, Fangyuan Shao, Xinhua Zhang, Chuxia Deng, Leli Zeng, Yulong He, Changhua Zhang

**Affiliations:** 1 Guangdong Provincial Key Laboratory of Digestive Cancer Research, Digestive Diseases Center, The Seventh Affiliated Hospital of Sun Yat-Sen University, Shenzhen, 518107 Guangdong China; 2 Shenzhen Key Laboratory of Chinese Medicine Active Substance Screening and Translational Research, Scientific Research Center, The Seventh Affiliated Hospital of Sun Yat-Sen University, Shenzhen, 518107 Guangdong China; 3 Guangdong-Hong Kong-Macau University Joint Laboratory of Digestive Cancer Research, Digestive Diseases Center, The Seventh Affiliated Hospital of Sun Yat-Sen University, Shenzhen, 518107 Guangdong China; 4 Shenzhen Key Laboratory for Drug Addiction and Medication Safety, Department of Ultrasound, Peking University Shenzhen Hospital, Shenzhen, 518036 China; 5 MOE Frontiers Science Center for Precision Oncology, Faculty of Health Sciences, Institute of Translational Medicine, Cancer Center, University of Macau, Macau SAR, 999078 China; 6 Department of Gastrointestinal Surgery, The First Affiliated Hospital of Sun Yat-Sen University, No. 58 Zhongshan Road, Guangzhou, 510080 China

**Keywords:** Gastrointestinal cancers, Gastrointestinal stromal tumor, Resistance, Immunology, Immunotherapy, Imatinib, Model, Immune checkpoint inhibitors

## Abstract

Mesenchymal gastrointestinal cancers are represented by the gastrointestinal stromal tumors (GISTs) which occur throughout the whole gastrointestinal tract, and affect human health and economy globally. Curative surgical resections and tyrosine kinase inhibitors (TKIs) are the main managements for localized GISTs and recurrent/metastatic GISTs, respectively. Despite multi-lines of TKIs treatments prolonged the survival time of recurrent/metastatic GISTs by delaying the relapse and metastasis of the tumor, drug resistance developed quickly and inevitably, and became the huge obstacle for stopping disease progression. Immunotherapy, which is typically represented by immune checkpoint inhibitors (ICIs), has achieved great success in several solid tumors by reactivating the host immune system, and been proposed as an alternative choice for GIST treatment. Substantial efforts have been devoted to the research of immunology and immunotherapy for GIST, and great achievements have been made. Generally, the intratumoral immune cell level and the immune-related gene expressions are influenced by metastasis status, anatomical locations, driver gene mutations of the tumor, and modulated by imatinib therapy. Systemic inflammatory biomarkers are regarded as prognostic indicators of GIST and closely associated with its clinicopathological features. The efficacy of immunotherapy strategies for GIST has been widely explored in pre-clinical cell and mouse models and clinical experiments in human, and some patients did benefit from ICIs. This review comprehensively summarizes the up-to-date advancements of immunology, immunotherapy and research models for GIST, and provides new insights and perspectives for future studies.

## Introduction

Mesenchymal gastrointestinal cancers are represented by the gastrointestinal stromal tumors (GISTs) which occur throughout the whole gastrointestinal tract, and affect human health and economy globally [1]. The annual incidence of GIST worldwide is about 10–15 per million [2], which is much higher in East Asia than that as compared to North America [2, 3]. About 20–30% of GIST patients exhibit malignant behaviors [4], and the five-year survival rate of malignant GIST patients is around 35–65% [4], which seriously threatens human health.

**Fig. 1:**
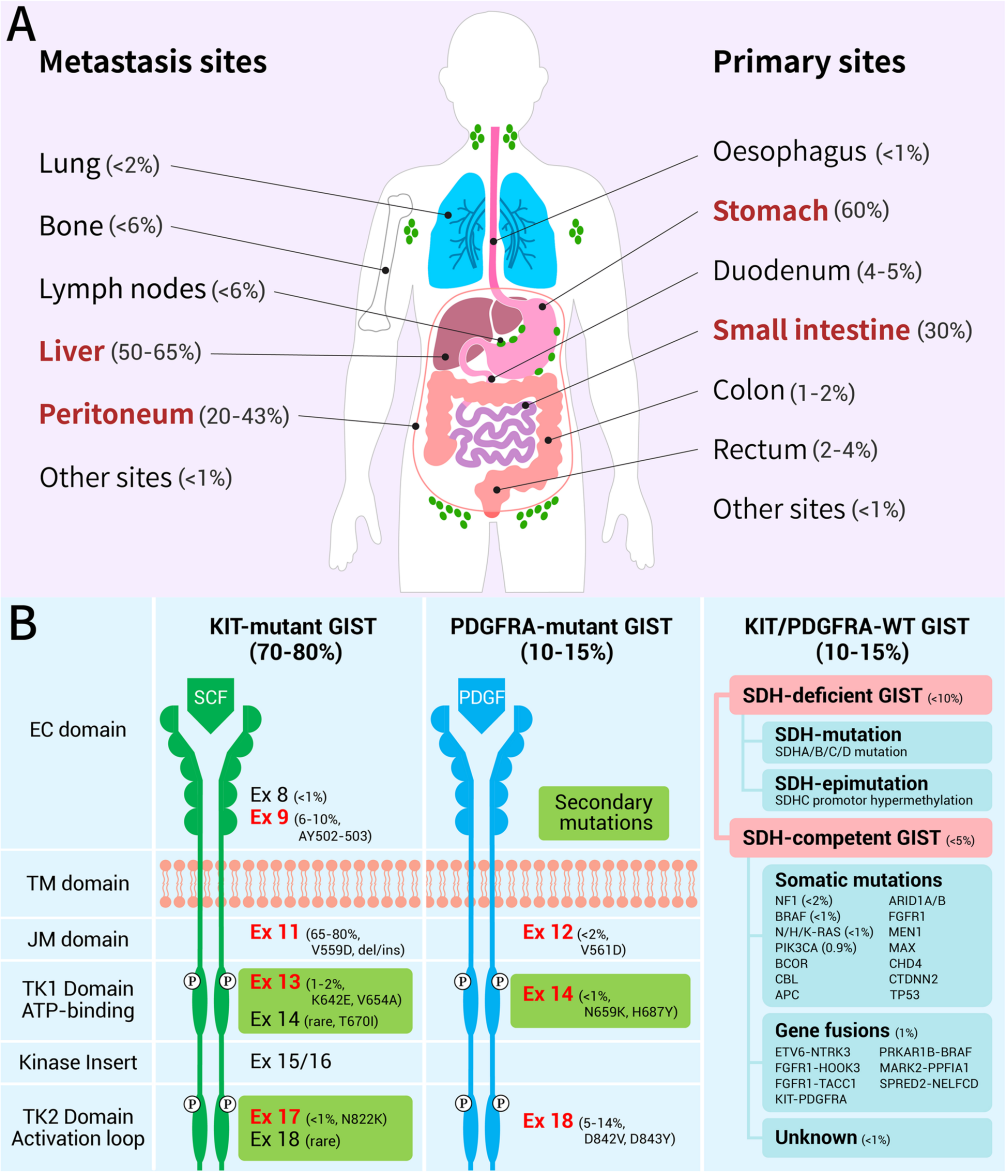
The anatomic and genomic distribution of GIST. **A** The anatomic locations of primary and metastatic GISTs. Primary GISTs usually originate from stomach and small intestine, and metastasize to liver or peritoneum. **B** The genomic profiles of GISTs. KIT and PDGFRA signaling are activated by their natural ligands SCF and PDGF respectively in physiological conditions, but are constitutively activated by oncogenic mutations and in a ligand independent manner in GISTs. According to the driver mutations, GISTs can be broadly classified as KIT-mutant GISTs, PDGFRA-mutant GISTs and KIT/PDGFRA-WT GISTs, and the last one could be further divided into SDH-deficient and SDH-competent GISTs based on the expression of succinate dehydrogenase. GIST: gastrointestinal stromal tumor; PDGFRA: platelet-derived growth factor receptor alpha; WT: wild type; EC: ligand-binding extracellular domain; TM: transmembrane domain; JM: intracellular juxtamembrane domain; TK: tyrosine kinase domain; SDH: succinate dehydrogenase; SCF: stem cell factor; PDGF: platelet-derived growth factor

Although primary GISTs can occur anywhere throughout the gastrointestinal tract (Fig. 1A), most of them originate from the stomach (60%) and small intestine (30%), and they can also be found in duodenum (4–5%), rectum (2–4%), colon (1–2%) and oesophagus (< 1%) [5–7]. Extragastrointestinal stromal tumor (eGIST) is extremely rare. Metastasis is common in advanced GIST which usually metastasized to liver (50–65%) and peritoneum (20–43%), and less frequently to lymph node (< 6%), bone (< 6%) and lung (< 2%), with other sites relatively rare [8–10].

GISTs are widely recognized to originate from the interstitial cells of Cajal (ICCs), the pacemaker cells located within the gastrointestinal wall, and are mainly caused by driver gene mutations. Mutations of KIT and platelet-derived growth factor receptor alpha (PDGFRA), which cause the constitutive activation of the KIT and PDGFRA signaling pathway, respectively, in a ligand-independent manner, are the major molecular mechanisms of the occurrence and progression of GIST [11, 12]. Both KIT and PDGFRA are homologous type III receptor tyrosine kinases consisting of five immunoglobulin (Ig)-like domains (Fig. 1B), namely ligand-binding extracellular domain (EC), transmembrane domain (TM), intracellular juxtamembrane domain (JM) and two tyrosine kinase domain (TK) [13]. According to the driver genes’ mutations, GISTs can be grossly divided into KIT-mutant GISTs, PDGFRA-mutant GISTs and KIT/PDGFRA-wild type (WT) GISTs (Fig. 1B).

About 70–80% of GISTs have KIT gene mutations [14–16] with the most common mutation sites on exon 11 (65–80%) and exon 9 (6–10%), followed by exon 13 (1–2%), exon 17 (< 1%) and exon 8 (< 1%) [14–16]. Around 10–15% of GISTs have PDGFRA gene mutations [14–16] with the common mutation sites on exon 18 (5–14%), exon 12 (< 2%) and exon 14 (< 1%) [14–16]. The other GISTs without KIT or PDGFRA mutations (10–15%) are defined as KIT/PDGFRA-WT GISTs, which include succinate dehydrogenase-deficient GIST (SDH-deficient GIST) (< 10%) that caused by SDHA/B/C/D mutations or SDHC promotor hypermethylation modifications, and succinate dehydrogenase-competent GIST (SDH-competent GIST) (< 5%) that caused by other somatic gene mutations or gene fusions, such as neurofibromatosis type 1 (NF1) (< 2%), B-raf proto-oncogene (BRAF) (< 1%) and rat sarcoma viral oncogene (RAS) (< 1%) gene mutations, and other GISTs with unknown mutations [15, 17, 18].

Before the approval of imatinib in the treatment of GIST, GIST was known to be insensitive to traditional chemotherapies [19–21], and the management of primary localized GISTs relied heavily on surgical resections. However, about 40–50% of high-risk GIST patients developed local recurrences or metastatic diseases within 5 years after the surgery [8, 22, 23] and their prognosis was very poor with the median overall survival (mOS) of only 12–19 months [8]. In 2002, imatinib was approved for the treatment of recurrent/metastatic or unresectable GISTs and quickly became the standard first-line therapy which dramatically improved the median progression-free survival (mPFS) and mOS [24, 25]. Unfortunately, not all GIST patients benefited from imatinib therapy, those with KIT/PDGFRA-WT genotype and PDGFRA-D842V mutations responded poorly to the treatment. In addition, due to secondary mutations of KIT, most of the imatinib-sensitive patients developed resistance to imatinib within 2 years [26–28]. Recently, the Food and Drug Administration (FDA) of the United States approved sunitinib, regorafenib and ripretinib as the second-, third- and fourth-line drugs for GIST, respectively, which prolong the mPFS of metastatic or recurrent patients with 5.6 [29], 4.8 [30] and 6.3 [31] months, respectively. Avapritinib was also recently approved by the FDA, and the mPFS is 29.5 months in metastatic PDGFRA-D842V GIST patients [32], and 3.7 months in non-D842V GIST patients [33].

Certainly, the discovery of imatinib and the successive tyrosine kinase inhibitors (TKIs) in the past two decades have revolutionized the management of recurrent/metastatic or unresectable GISTs and provided additional survival benefit for patients by delaying metastasis and recurrence. However, due to the primary and secondary resistance, TKIs often have short-lived disease control and very limited efficacy, and thus most GIST patients ultimately became refractory to these treatments. Therefore, novel therapeutic targets and drugs are urgently necessary to circumvent the resistance and further improve treatment efficacy. Fortunately, immune checkpoint inhibitors (ICIs) have achieved great success recently in various malignant tumors, and some patients can even be cured. GIST microenvironment has been demonstrated to be populated by a large amount of tumor-infiltrating immune cells, which play an essential role in tumor surveillance and may be exploited to remove imatinib resistant tumor cell clones, thereby enhancing the antitumor effect of imatinib. Unfortunately, in early phase trials, heavily pretreated GIST patients only showed moderate responses to therapeutic antibodies against programmed cell death protein 1 (PD1) and cytotoxic T-lymphocyte antigen 4 (CTLA-4) [34, 35], and the mechanisms of these treatments are obscure. This article will systematically review the research progress of immuno-oncology, immunotherapy and research models of GIST, and discuss the difficulties encountered in immunotherapy, so as to provide new insights into the development of more effective immunotherapies for GIST.

## Systemic inflammation in GIST

Inflammation plays a vital role in tumorigenesis, tumor progression, invasion, metastasis and angiogenesis [36]. Therefore, tumor‐promoting inflammation is recognized as one of the hallmarks of cancers [37]. The malignant phenotypes of tumor cells stimulated the infiltration of inflammatory cells [38], while the destruction of tumor cells via physical or chemical strategies usually caused the generalized and nonspecific systemic inflammatory responses which were characterized by thrombocytosis, neutrophilia, lymphocytopenia [39]. Increasing evidence suggested that some hematological biomarkers based on the number of blood leukocytes and platelets, for example, NLR, PLR, PNI, MLR, NWR, MWR, LWR, PWR, NAR, LMR, HALP, SII and so on (the meanings of these abbreviations were presented in Table 1), reflecting the systemic inflammatory response of the host. Given that these systemic inflammatory biomarkers are accessible, reproducible and cost-effective, and are associated with patients’ prognosis of various kinds of cancers, they are often used to predict the survival outcome and treatment responses, as well as to help clinicians to determine appropriate therapeutic schemes for patients [40, 41]. In GIST, several inflammatory hematological biomarkers, which are detailed in Table 1, has been proven to be closely related to the clinical outcomes and clinicopathological features.

**Table 1:** Systemic inflammatory indicators in GIST

Author & Year	Regions	No. of patients	Sample	Tested items	Prognosis	Associated clinicopathological parameters	Ref
Perez et al. 2013	USA	339	Preoperative	NLR	RFS (-)	Tumor size, mitotic index	[42]
Jin et al. 2013	China	42	Preoperative	NLR	OS (-)	Tumor size, mitotic index	[43]
Atila et al. 2014	Turkey	67	Preoperative	NLR	DFS (-)	NA	[44]
Kargın et al. 2015	Turkey	78	Preoperative	NLR	OS (-)	Mitotic index	[45]
Racz et al. 2015	Canada	93	Preoperative	PLR, neutrophils	RFS (-)	Tumor size, mitotic index	[39]
Goh et al. 2016	Singapore	300	Preoperative	NLR, PLR	RFS (-)	NIH risk, AFIP risk	[46]
Stotz et al. 2016	Austria	149	Preoperative	PLR	RFS (-)	NA	[47]
Jiang et al. 2016	China	129	Preoperative	NLR	OS (-)	Tumor stage	[48]
Feng et al. 2016	China	274	Preoperative	NLR, MLR, PLR, NWR, MWR, low LWR	RFS (-)	Tumor size and location, mitotic index, NIH risk	[49]
Yin et al. 2017	China	400	Preoperative	PLR	RFS (-)	Tumor size, mitotic index, NIH risk	[50]
Xue et al. 2017	China	510	Preoperative	NLR	RFS (-)	NA	[51]
Luo et al. 2018	Meta	1676	Unclear	NLR	DFS (-), RFS (-)	Tumor size, mitotic index, NIH risk	[52]
Hu et al. 2018	China	92	Preoperative	PLR	RFS (-)	Mitotic index	[53]
Rutkowski et al. 2018	Poland	385	Imatinib treated	NLR	OS (-), RFS (-)	Mitotic index, driver mutation	[54]
Liu et al. 2018	Meta	1735	Preoperative	PLR	DFS (-), RFS (-)	Tumor size, mitotic index, NIH risk	[55]
Zhang et al. 2019	Meta	2264	Unclear	NLR	DFS (-), RFS (-)	Not mentioned	[56]
Yilmaz et al. 2019	Turkey	45	Unclear	NLR	OS (-)	NA	[57]
Sun et al. 2019	China	431	Preoperative	NLR, PLR	RFS (-)	NA	[58]
Sobczuk et al. 2019	Poland	146	Preoperative	NLR	OS (-)	Not associated	[59]
Yang et al. 2019	China	72	Preoperative	NLR	OS ( +)	Tumor size and location, age	[60]
Cananzi et al. 2019	Italy	127	Preoperative	MLR, NLR, PLR	DFS (-)	NA	[38]
Shi et al. 2019	China	340	Preoperative	PNI	RFS ( +)	NIH risk	[61]
Sun et al. 2020	China	85	Preoperative	NLR	PFS (-), OS (-)	NA	[62]
Wei et al. 2020	Meta	3135	Unclear	NLR, PLR	DFS (-)	Tumor size, tumor stages, mitotic index	[63]
Catal et al. 2020	Turkey	30	Preoperative	LMR	NA	AFIP risk score	[64]
Lin et al. 2020	China	424	Preoperative	PLR	RFS (-)	Not associated	[65]
Li et al. 2020	China	229	Unclear	NAR	RFS (-)	Not associated	[66]
Chang et al. 2020	China	646	Preoperative	PLR	DFS (-), OS (-)	Tumor size and location, NIH risk	[67]
Guo et al. 2020	China	143	Preoperative	Lymphocyte	DFS ( +)	No significant association	[68]
Yan et al. 2021	China	843	Preoperative	NLR, WLR, MLR, PLR	NA	NIH risk	[69]
Li et al. 2021	China	392	Preoperative	PNI	RFS ( +)	NA	[70]
Lu et al. 2021	China	160	Preoperative	SII	RFS (-)	NA	[71]
Zhao et al. 2022	China	591	Preoperative	HALP	RFS ( +)	Tumor size, site, NIH risk, mitotic index	[72]
Li et al. 2022	Meta	2627	Preoperative	PNI	RFS ( +)	NA	[73]
Yang et al. 2022	China	455	Preoperative	CONUT	RFS (-)	Tumor size and location	[74]
Ding et al. 2023	China	57	Preoperative	SII-PNI	RFS (-)	NA	[75]

*Abbreviations DFS* Disease-free survival, *RFS* Recurrence-free survival, *OS* Overall survival, *PFS* Progression free survival, *PLR* Platelet-to-lymphocyte ratio, *NLR* Neutrophil-to-lymphocyte ratio, *MLR* Monocyte-to-lymphocyte ratio, *NWR* Neutrophil-to-white blood cell ratio, *LWR* Lymphocyte-to-white cell ratio, *MWR* Monocyte-to-white cell ratio, *PWR* Platelet-to-white cell ratio, *NAR* Neutrophil-to-albumin ratio, *LMR* Lymphocyte-to-monocyte ratio, *AFIP* Armed Forces Institute of Pathology, *HALP* The combined index of hemoglobin, albumin, lymphocyte, and platelet, *PNI* Prognostic nutritional index (albumin plus lymphocytes), *SII* Systemic immunoinflammatory index (platelet count × neutrophil count/lymphocyte count), *CONUT* Controlling nutritional status calculated from albumin, lymphocyte count and cholesterol, *SII-PNI* SII combined with PNI scores. + , positive correlation; -, negative correlation

To date, the underlying mechanisms of systemic inflammation in promoting tumor progression and influencing the prognosis of cancer patients remain to be elucidated [60, 72]. Several potential mechanisms have been proposed and may help to explain some clinical observations. *First*, thrombocytosis is associated with poor prognosis of cancer patients. As a reservoir of secreted proteins, platelets in the blood are able to secrete a variety of growth factors, cytokines and chemokines, which in turn, promote tumor growth, survival, metastasis and angiogenesis [76]. Moreover, platelets have also been demonstrated to infiltrate into tumor microenvironment and directly interact with tumor cells [77], to help circulating tumor cells to adhere to endothelial cells, and thereby to establish a niche prior to metastasis [78]. *Second*, peripheral neutrophil is an indicator for acute and chronic inflammation [79]. On the one hand, neutrophil promotes angiogenesis and progression of tumors [79] through secreting tumor growth promoting factors, such as vascular endothelial growth factor (VEGF) [80, 81], matrix metalloproteinase [82, 83], hepatocyte growth factor (HGF) [84], interleukin 6 (IL-6) [85] and IL-8 [86]. On the other hand, high neutrophil counts suppress immune system of the host via restraining the cytolytic activity of immune cells, including lymphocytes, activated T cells and natural killer cells [87]. In addition, neutrophil has also been found to promote tumorigenesis by inhibiting the functions of T cells through reactive oxygen species (ROS) and arginase-1 [88, 89]. *Last but not least*, lymphocytes are crucial for the cell-mediated antitumor immune response. Lymphocytes inhibit the proliferation and metastasis of tumor cells by inducing cytotoxic cell death and cytokine secretion [90]. Peripheral lymphocytes are closely related to tumor-infiltrating lymphocytes (TILs); the lower the circulating lymphocytes count, the lower the infiltrating lymphocytes level, which eventually leads to the decreased antitumor activity and poor prognosis [91]. Therefore, the blood lymphocytes counts reflect the degree of responsiveness of the host to the clinical managements [92, 93].

## Immune cell landscape of GIST

**Fig. 2:**
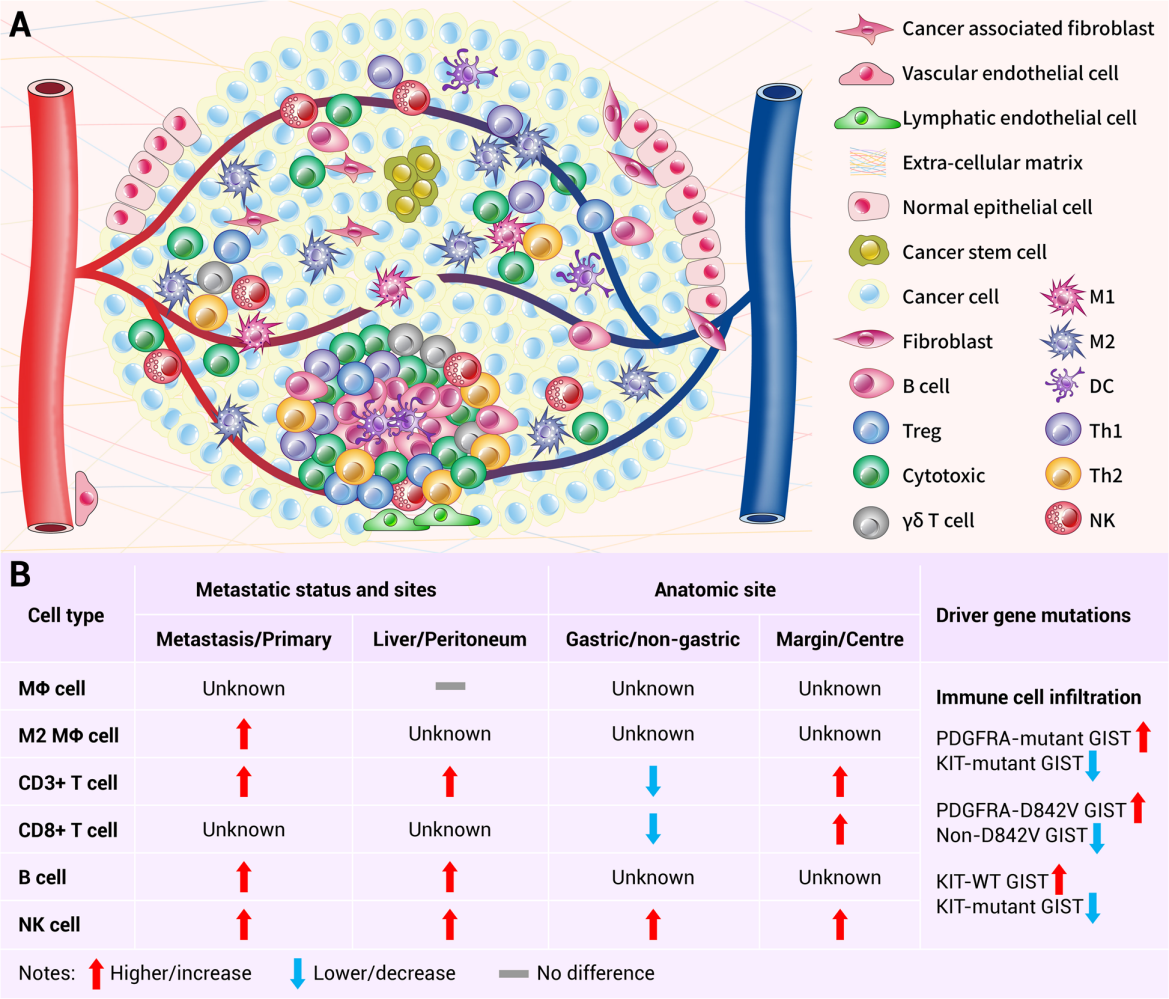
The immune microenvironment of GIST. **A** GIST is mainly infiltrated with T cells and M2 macrophages, and less frequently by NK, B and DC cells. Approximately half of the GISTs harbor intratumoral tertiary lymphoid structures which were enriched with T cells and B cells. **B** Gross comparisons of the immune cell infiltration between different groups. Immune cells seem to be enriched in metastatic GIST (especially liver metastasis), non-gastric GIST and PDGFRA-mutant GIST and the margin area of the GIST. GIST: Gastrointestinal stromal tumor; NK: natural killer cell; MΦ: macrophages; M1: M1 that macrophages are classically activated; M2: M2 macrophages that are alternatively activated; DC: dendritic cell; Th1: T helper type 1 cell; Th2: T helper type 2 cell; γδ T cell: gamma delta T cell with T cell receptors (TCRs) composed of γ- and δ-chains

Several studies have explored the immune landscape of GIST and found that almost all GIST samples are infiltrated with variable amounts of immune cells [94–96]. Macrophages and T lymphocytes are the most common immune cells in GIST [94, 95, 97–104] and are representatively illustrated in Fig. 2A; although some studies showed that the former is more abundant than the latter [97, 101, 104], others reported the opposite [94, 96, 98, 102, 105–108]. Such discrepancies may be explained by the different anatomical tumor sites [94, 109], metastatic status [97, 98, 101, 109–111] and driver gene mutations [99, 112]. Moreover, there are also some less abundant immune cells infiltrated in GIST, including natural killer (NK) cells, B lymphocytes, dendritic cells (DCs), natural killer T (NKT) cells, gamma delta (γδ) T cells, neutrophils, eosinophils and mast cells, etc. [42, 94, 96, 100, 101, 103, 106, 113–115]. The intratumoral distribution patterns of immune cells in GIST are variable; they are mainly diffusely distributed around blood vessels with a small amount distributed in aggregates [94, 96, 100, 101, 105]. Immune cells infiltrated in GIST have been shown to be associated with patients’ clinicopathological features, and have predictive values. Since DC cells [97], myeloid-derived suppressor cells (MDSCs) [97] and neutrophils [42] are rarely present and reported in GIST, this article will focus on the infiltration of macrophages, T lymphocytes, NK cells and B cells and their relationship of the clinicopathological characteristics of GIST patients.

### Tumor-associated macrophages

Tumor-associated macrophages (TAMs) are one of the most common inflammatory cells in the microenvironment of GIST [97], and can be classified as classically activated (M1) and alternatively activated (M2) macrophages. M1 macrophages exert antitumor activity through phagocytizing tumor cells, presenting tumor cell antigens to T cells and producing proinflammatory cytokines; on the contrary, M2 macrophages, by suppressing inflammatory responses, recruiting Treg cells, and stimulating angiogenesis, promote the tumor progression. In untreated primary GIST, the polarization of TAMs in the microenvironment is controversial, with either M2 [102] or M1 macrophage [116] considered to be the major cell subtypes. Whereas in metastatic and imatinib-treated GISTs, M2 macrophage is the most enriched subtype [97, 113, 116, 117] with high expression level of major histocompatibility complex class II (MHC-II) molecules [97]. Previous studies have demonstrated that the amount of CD68^+^ macrophages (both M1 and M2) was positively correlated with RFS [118, 119] and risk grade of GIST [94, 95], but the ratio of CD163^+^ macrophages (M2 only) to CD68^+^ macrophages showed a negative correlation with RFS [118, 119]. However, some studies reported that there were no significant correlations between macrophages counts and RFS [106], or the prognosis [94] of GIST. The M2 macrophage was positively associated with the expression of ETS variant 1 (ETV1) which was highly expressed on GIST cells, and ETV1 inhibition was found to depress the malignant progression of GIST via suppressing the M2 polarization of macrophages [120]. Li et al. identified a group of macrophage-like circulating tumor cells (ML-CTC) [121, 122]featured by the expression of both CD68 (macrophage cell marker) and KIT (GIST cell marker) in metastatic GIST, and proposed that these cells may be used to predict the relapse and metastasis of GIST in future studies [122].

### Tumor-infiltrating T lymphocytes

T lymphocytes in GISTs are mainly CD3^+^ T cells, including CD8^+^ T, CD4^+^ T and a small amount of Foxp3^+^ T-regulatory (Treg) cells [97, 99, 123]. CD3^+^ T cells in GIST have been demonstrated to be highly activated and enriched in MHC-I positive regions of tumors [99]. CD3^+^ T cells populated in GIST showed a negative correlation with the tumor size [99], positive correlations with RFS [99, 106, 109], improved PFS [99] and OS [109], and thus CD3^+^ T cell infiltration possess certain prognostic values. While, some other studies demonstrated controversial results, Cameron et al. reported that the infiltration of CD3^+^ T cells was positively correlated with cell proliferation index [101] and high risk GISTs [101], and was not associated with metastasis [124] or survival [125] in GIST.

CD8^+^ T lymphocytes are the key lymphocytes to kill cancer cells and to achieve a response to anti-PD1 antibody treatment [126]. GISTs have been found to harbor a high infiltration level of CD8^+^ T cells [127], and nearly all GIST samples have CD8^+^ T cells [108], but the cell number in GIST is much lower than those in non-small cell lung cancer (NSCLC) [34]. CD8^+^ T lymphocytes in GIST have been shown to be positively correlated with RFS [106, 109, 118, 119, 128] and OS [109, 128]. In PD-L1^+^IDO^+^ GIST patients, CD3^+^CD8^+^ T lymphocytes were negatively correlated with the tumor size [124]. Dickkopf 4 (DKK4) is highly expressed in high-risk GISTs and negatively correlated with the number of intratumoral CD8^+^ T cells [129]; GIST cells directly inhibit the migration and infiltration of CD8^+^ T cells by secreting DKK4 [129]. As for CD4^+^ T cells, they are also infiltrated in nearly all GIST samples, but the number is less abundant than those of CD8^+^ T cells [108]. So far, the prognostic value of CD4^+^ T cell infiltration in GIST has not been well documented. In *Kit*^*V558Δ/*+^ mouse GISTs, γδ T cells were found to be present in GIST, accounting for about 2% of immune cells in GIST [115], and promote the antitumor immunity through IL17A secretion [115].

Treg cells, which inhibit antitumor immune response, are relatively rare in GIST [97], although seem to be higher than in other sarcomas [123]. Treg cells infiltrated in GIST showed a negative correlation with PFS [99] but a positive correlation with high risk GISTs [98, 99, 130], and no association between Treg cell infiltration and GIST metastasis was found [124]. In addition, the infiltration of Treg cells in GIST is positively correlated with that of M2 macrophages, suggesting that the immunosuppressive effect of the latter may be attributed to Treg cells recruitment [130]. The CD8^+^ T/Foxp3^+^ Treg cell ratio in GIST is much lower than in cervical cancer [131], which suggests a strong immune suppression in GIST microenvironment [97].

### Tumor-infiltrating NK cells

NK cells belong to the innate immune system and are the first-line defense against infection and tumors. They can target cells with low MHC-I expression, serving as an important supplement to the cell-mediated antitumor immunity. Different from other solid tumors [132, 133], NK cells are abundant in GISTs [99, 102, 108, 134] and are more likely to enrich in gastric GISTs [99]; around 42.1% of GISTs have CD56^+^ NK cells [108] and around 25% of CD45^+^ leukocytes are CD3^−^CD56^+^ NK cells [134]. NK cells infiltrated in GISTs are positively correlated with PFS [99, 128, 133], RFS [99, 106, 109], OS and prognosis [101, 109, 128], but negatively correlated with high risk GISTs [99], proliferation index [101, 134] and metastases [134]. In addition, the level of interferon γ (IFNγ), secreted by NK cells, was also positively associated with better survival of GIST patients [133, 135]. NK cells play an important role in the metastasis of GIST [136]. With the use of NK or T cell-depleting monoclonal antibodies on mouse model, it has been shown that NK cells exert antitumor activity mainly in the process of metastasis, but not in the primary tumors [137].

NK cells express several receptors, such as natural killer cell p30-related protein (NKp30) and NKp46, which endow NK cells with distinct functions. NKp30 has three major isoforms, namely NKp30A, NKp30B and NKp30C. NKp30A and NKp30B receptors mediate cytotoxicity and generation of IFNγ/tumor necrosis factor alpha (TNFα), respectively, whereas NKp30C induces the production of immunosuppressive cytokine IL-10 [132–134]. Compared with those from healthy volunteers, peripheral blood mononuclear cells (PBMCs) from GIST patients express lower levels of natural cytotoxicity triggering receptor 1 (NCR1, encoding NKp46), NKp30A and NKp30B, while the NKp30C level is comparable between these two groups [138]. In GIST, NK cells predominantly express the immunosuppressive NKp30C isoform [99, 134] whose expression level shows negative correlations with OS and prognosis [133, 134]. B7-H6, a ligand of NKp30, is widely expressed in GIST [99, 133, 134, 139]; its soluble form, sB7-H6, is negatively associated with DFS and prognosis in metastatic GIST [133]. Baculoviral IAP repeat-containing protein 3 (BIRC3) and Tumor necrosis factor receptor (TNFR) associated factor 1 (TRAF1) are highly expressed in NK cells, and their expressions are correlated with NKp30C level [140], suggesting that BIRC3 and TRAF1 are involved in the regulation of NK cell activity in GIST. In addition, the transcription of BIRC3 induced by TNFα reduces the expression of NKp46, the activation receptor [140], thereby weakening the antitumor effect of NK cells and promoting the metastasis and dissemination of GIST, resulting in poor prognosis [140]. The presence of membrane-bound transforming growth factor β (TGFβ) in Treg cells downregulates the expression of natural killer group 2 member D (NKG2D) receptor in NK cells, which directly inhibits the cytotoxicity of the latter [141]. Depletion of Treg cells in mouse exacerbates the proliferation and cytotoxicity of NK cells [141].

### Tumor-infiltrating B lymphocytes and tertiary lymphoid structures

The tumor-infiltrating B cells in tertiary lymph nodes are crucial in antitumor immune responses; they contribute to humoral antitumor responses through antibody-dependent cellular cytotoxicity (ADCC) and complement-dependent cytotoxicity (CDC). B lymphocytes are present in GIST [94, 105] and may be more prevalent in isolated intratumoural lymphoid aggregates [96]. B lymphocytes are negatively correlated with the tumor size [105] but positively correlated with RFS [106, 109] and OS [109] in GIST. However, Cameron et al. reported the opposite findings showing that the B lymphocytes are less infiltrated in GIST and mainly located in metastatic GIST, which showed positive correlations with cell proliferation index, recurrence risk and metastasis [101].

Tertiary lymphoid structures (TLSs), which usually comprise a T cell zone and a B cell follicular zone, are ectopic lymphoid aggregates that are widely present in various cancer types [142]. As diagrammed in Fig. 2A, Tumor-infiltrating TLSs were found in 44.9%-52.2% GISTs [143], and their outer layers were mainly composed of CD4^+^ T and CD8^+^ T cells while the inner layers were mostly composed of B cells [143]. TLSs were found to be associated with low risk GISTs, longer survival time, RFS and lower imatinib resistance [143], and thus may be a novel therapeutic strategy for imatinib-resistant GIST patients [143]. No difference was found in the morphology of TLSs between different types of GISTs with various driver gene mutations [143], but PDGFRA-mutant GISTs were more likely to have TLSs when compared to KIT-mutant GISTs and WT GISTs [143]. Further analysis showed that TLS^+^ PDGFRA-mutant GIST patients had the best survival outcome while TLS^−^ KIT-mutant patients had the worst OS [143].

### Immune cell infiltration and metastasis

As shown in Fig. 2B, The infiltration level of immune cells is closely related to the metastatic status of GIST [111]. In primary GISTs, the amount of CD68^+^ macrophages is higher than that of CD3^+^ T-cells, whereas the opposite is the case in metastatic GISTs [101]. Metastatic GISTs harbor more M2 macrophages [111], which are approximately twice as many as those of primary GISTs [97], suggesting that M2 macrophages promote the tumor progression. Moreover, the numbers of infiltrated CD3^+^ T [101], B [101, 110] and NK cells [101, 110] are also much higher in metastatic GISTs than in primary GISTs [101]. At the tumor margin, local non-metastatic GISTs exhibit more CD8^+^ T and Foxp3^+^ Treg cells than metastatic GISTs [109]. In addition, the infiltration levels of immune cells are also closely associated with the metastatic sites of GIST. When compared with peritoneal metastases, liver metastases have more CD3^+^ T, CD56^+^ NK and CD20^+^ B cells, but CD68^+^ macrophages are comparable [101].

### Immune cell infiltration and anatomic sites

The infiltration levels of immune cells are related to the anatomic sites of GISTs as different lesions have distinct microenvironment (Fig. 2B). Gastric GISTs possess the most immune cell infiltration while eGISTs have the least infiltration [94]. When compared with non-gastric GISTs, gastric GISTs harbor less CD8^+^ T [103, 109] and CD3^+^ T cells [101] but more NKp46^+^ NK cells [99]. Besides, more CD8^+^ T, CD3^+^ T, CD4^+^ T and NKp46^+^ NK cells are found at the edge of tumors compared to tumor centers [109].

### Immune microenvironment and driver gene mutations

The infiltration of immune cells and the expression of immune-related genes differ between GISTs driven by different mutations, and are grossly illustrated in Fig. 2B. The infiltration levels of immune cells of KIT/PDGFRA-WT GISTs are still controversial. Sun et al. reported that KIT/PDGFRA-WT GISTs had more CD8^+^ T cells than KIT- and PDGFRA-mutant GISTs [103]; however, Gasparotto et al. reported the opposite which showed that the former had less T cells, including CD3^+^ T, CD4^+^ T and CD8^+^ T cells [104], and expressed lower levels of MHC-I and immune checkpoint molecules [104].

When compared with KIT-mutant GISTs, PDGFRA-mutant GISTs harbor a higher infiltration level of immune cells [104, 112], especially that of CD8^+^ T cells, and display a higher cytolytic activity [112]. PDGFRA-mutant GISTs highly express C-X-C motif chemokine ligand 14 (CXCL14) [112] and enhance immune surveillance by recruiting DC, NK and CD8^+^ T cells and upregulating MHC-I molecules levels [144]. Moreover, PDGFRA-mutant GISTs increase the expression of C–C motif chemokine ligand 2 (CCL2) through NF-kB (nuclear factor kappa-light-chain-enhancer of activated B cells) signaling [104], which helps to recruit macrophages to GIST microenvironment [145]. Given that PDGFRA-mutant GISTs have the strongest immune-related gene expression signatures, exhibit more neoepitopes that could be presented by MHC-I molecules, express more immune checkpoint molecules, and possess higher immunogenicity, they may respond better to immunotherapy [112], and these features may also at least partially explain why PDGFRA-mutant GISTs have a more favorable disease outcome [112, 146–148].

PDGFRA-D842V mutation, which hampers the binding of imatinib via changing the conformation of the kinase domain of PDGFRA [149, 150], is the most common genetic mutation leading to the primary resistance to imatinib. Many research studies have shown that PDGFRA-D842V GISTs have more immune cells infiltration and higher cytolytic activity than non-D842V GISTs [98, 112, 149, 151]. Moreover, PDGFRA-D842V GISTs also express higher levels of interferons and chemokines, as well as PD1 and programmed death-ligand 1 (PD-L1), and has more driver-derived neoepitopes that could be presented by MHC-I molecules [98, 112, 149].

Until now, limited research studies have examined the impact of KIT mutational subtypes on GIST microenvironment. In KIT-mutant GISTs, intestinal GISTs have been shown to have more immune cells infiltration than non-intestinal GISTs [104]. In localized GISTs, it was found that the number of NK cells in KIT-WT GISTs is threefold higher than that in KIT-mutant GISTs (with Exon-11 mutation) [99].

## Immune checkpoint, MHC and other immune related genes

### PD1/PD-L1

PD1 and its ligand PD-L1 are expressed on the surface of immune cells and tumor cells, respectively; the binding of PD-L1 to PD1 causes inhibition or diminution of the immune cell function, which, in turn, leads to immune escape and promotes tumor progression.

In GIST, PD-L1 is mainly present on tumor cells and a few in leukocytes [35], independent of the mutant types of the driver genes [35]. Its expression shows high heterogeneity in GIST [35, 152]; about 16.3%-69.0% of GIST samples present PD-L1 expression [34, 96, 98, 103, 105, 109, 123, 124], and in the same sample, there might be less than 10% of tumor cells express PD-L1 [34]. Overall, the expression level of PD-L1 in GIST is relatively low [34, 35, 104, 123], though it is higher than that in other kinds of sarcoma [123]. PD-L1 expression has been demonstrated to be correlated with clinicopathological features with predicting values [103, 152], but the results are controversial. Some studies showed that PD-L1^+^ GISTs have more immune cells infiltration [103, 105, 125], and PD-L1 expression in GIST was negatively correlated with tumor size [98, 103], mitotic index [103], high risk GISTs [152] and metastasis [152], and was positively correlated with improved RFS [103], suggesting that high PD-L1 expression is associated with antitumor immune response and better prognosis. However, other studies found that PD-L1 expression was positively correlated with the features of unfavorable outcomes, such as tumor size [124], proliferation index [124], high-risk GIST [125, 153] and therapy resistance [153], but was not associated with RFS [109], metastasis [124] and OS [105, 109, 124]. Besides, the expression level of PD-L1 on immune cells is related to worse DFS [154, 155]. In plasma, high expression of soluble PD-L1 (sPD-L1) is associated with PFS and poor prognosis [156–159].

PD1 is primarily expressed on GIST-infiltrated T cells [35] with low levels [104, 127]. PD1 has been reported to be present on 48.5% or 88% of GIST samples [98, 103], and such discrepancy may be associated with the samples they chose. The expression of PD1 in intratumoral T cells is higher than that in circulating T cells [35] and its expression shows no correlation with RFS or OS [109]. Same as sPD-L1, the high expression of soluble PD1 (sPD1) in plasma is associated with PFS and poor prognosis [156–158].

### IDO

Indoleamine 2,3-dioxygenase (IDO), the rate-limiting enzyme of tryptophan metabolism in human, metabolizes tryptophan, an essential amino acid, into kynurenin, which changes the tumor microenvironment from immunogenic to tolerogenic [160]. IDO exerts immunosuppressive effects by directly inhibiting the CD8^+^ T cell activity and inducing Treg cell differentiation [161–163]. In addition, tryptophan metabolites are able to polarize antigen presenting cells (APCs) to exhibit an immunotolerant phenotype featured by secreting TGFβ or IL-10 [164]. In GIST, the constitutively activated KIT signaling upregulates IDO expression through the transcription factor ETS variant 4 (ETV4) [95]. The IDO expression level is high in GIST with 63%-89.8% of GISTs are IDO-positive [34, 124] and almost all of PD-L1^+^ GISTs are IDO^+^ GISTs [124]. Moreover, all PDGFRA-mutant GISTs express IDO but show no correlation with clinicopathological features [98]. In addition, CD4^+^ T cells are more abundantly infiltrated in PDGFRA-mutant GISTs with high IDO level [98], while in PD-L1^+^IDO^+^ GIST, higher infiltration level of Treg cells is found [124]. In *Kit*^*V558Δ/*+^ GIST mice, IDO inhibitors may enhance the antitumor effect of imatinib [95] or anti-PD1 antibody [35] through activating CD8^+^ T cells and inducing apoptosis of Treg cells [95, 165], suggesting that the IDO-targeted immunotherapy would be of great value.

### Tim-3/Gal-9 and other immune checkpoints

In GIST, T cell immunoglobulin and mucin-domain containing-3 (Tim-3) is mostly present in TILs [108] with low expression level [127]. Galectin-9 (Gal-9), the ligand of Tim-3, is expressed mainly in tumor cells [108]. Nearly all GIST samples with Tim-3^+^ NK-infiltration showed the Gal-9 expression [108], suggesting that their interactions are likely involved in the suppression of antitumor immunity, and therefore, blocking Tim-3/Gal-9 pathway may become a new strategy for GIST treatment [108]. The expression level of Tim-3 in GIST shows positive correlations with OS, PFS and density of CD8^+^ T cells [128], but a negative correlation with high risk GISTs [128]. Contrary to Tim-3, Gal-9 expression is positively correlated with high-risk GISTs [128], negatively correlated with the densities of CD8^+^ T cells and CD56^+^ NK cells [128], and displays no correlation with OS and PFS [128]. These conflicting findings suggest that Tim-3 and Gal-9 may have different mechanisms in terms of immune escape in GIST, which differ from those in epithelial tumors [128].

Lymphocyte activation gene-3 (LAG3) and V-type immunoglobulin (Ig) domain-containing suppressor of T-cell activation (VISTA) are mainly expressed on the surface of T cells. The expression of LAG3 in GIST is also low [127], but its expression in intratumoral T cells is significantly higher than that in circulating T cells [35]. The expression of VISTA in GIST is associated with improved outcomes [154, 155], which implies that VISTA has multifaceted roles in different cancers, and also highlights the complexity of VISTA as an immune checkpoint protein. In addition, inducible T cell costimulator (ICOS) and its ligand, B7H2 (ICOSL), are also present in GIST, and related to poor prognosis [166]. B7-H6, a novel immune checkpoint molecule, is able to elicit NK cells’ antitumor immune responses upon interacting with its receptor NKp30 on NK cells. Given B7-H6 is highly expressed in GIST [99, 134, 167], it will be a potential immunotherapy target.

### CTA, MHC-I and MICA/B

Cancer testis antigens (CTAs) are a large class of tumor-associated antigens, when presented by MHC-I molecules expressed on the surface of APCs, they can be recognized by specific cytotoxic T lymphocytes and trigger the antitumor immunity. CTAs are expressed in various malignant tumors, whereas in normal tissues, CTAs are only present in germ cells of the testis and placenta; therefore, CTAs are considered to be ideal targets for the immunotherapy of various cancers [168]. It has been reported that 26.7%-40% of GIST patients show CTAs expression [168–170]. G antigen (GAGE) [171], melanoma-associated antigen (MAGE)-A1 [168, 169], MAGE-A3 [168, 169], MAGE-A4 [168, 169], MAGE-C1 [168, 169], MAGE-C2 (CT10) [171] and New York esophageal squamous cell carcinoma-1 (NY-ESO-1) [168, 169] are present in 12%, 9%-14.3%, 8%-14.3%, 13%-14.3%, 15%-25.8%, 10% and 12%-20.0% of GIST patients, respectively. Another two studies, however, have demonstrated that NY-ESO-1 was almost not expressed in GIST [172, 173]. The CTAs expression is associated with clinicopathological features of GIST with predicting values [168, 169, 171]. CTA^+^ GISTs have worse responses to imatinib and shorter RFS [168, 169]; while the MAGE-A3 and NY-ESO-1 levels correlate with tumor progression after imatinib treatment [168].

MHC molecules, also called human leukocyte antigens (HLAs), are mostly expressed on the surface of tumor cells and antigen-presenting cells. However, only 30% of GIST samples show a normal expression level of MHC-I molecules [97], and a majority of them have partial defects of HLA expression; 38% of GISTs show no HLA-A expression and 20% show no expressions of HLA-B and HLA-C [97]. These findings suggest that there are interactions between tumor cells and immune cells and those clones with low levels of MHC-I molecules are selected and survive [97]. Defective expression of MHC-I molecules led to decreased recognition of tumor cells by cytotoxic T lymphocytes and weakened the antitumor immune responses [97]. Although the MHC-I expression is lost in GIST, MHC class I chain-related protein A and B (MICA/B) are found in GIST, suggesting that NK cells may play a crucial role in the antitumor immune responses [174, 175].

### Other immune-related genes

By releasing a variety of molecules, tumor cells are able to recruit different types of cells into tumor tissues, transform the tumor microenvironment and promote their growth and metastasis in return. The expression of inflammatory cytokines in GIST is very low, and the TNFα expression is basically negligible [100], suggesting an immunosuppressive microenvironment in GIST. As for chemokines, such as CCL2, CCL3 and CXCL1, their expressions in GISTs are relatively high [101] and CCL2 induces the infiltration of macrophages into tumor tissues and promotes tumor growth [101, 145]. CXCL2, which is mainly produced by M2 macrophages in metastatic GISTs, promotes migration, invasion and EMT of GIST cells in vitro and in vivo [117]. GIST cells facilitate p65 phosphorylation and nuclear translocation through lowering the expression of secreted protein acidic and rich in cysteine-like 1 (SPARCL1), and thereby increasing the release of cytokines and the infiltration of M2 macrophages [176]. In addition, GIST microenvironment contains large amounts of TGFβ1, which reduces the activity of immune effector cells [177] and promotes tumor metastasis. CC chemokine receptor type 8 (CCR8) is mainly present in Treg cells and negatively correlated with patients’ survival [178]. The ligand of CCR8, CCL1, enhances tumor immunosuppressive microenvironment via the recruitment of CCR8^+^ Treg cells [179]. Anti-CCR8 antibody is able to selectively eliminate the clonally expanding Treg cells within the tumor, but has no effect on tumor-infiltrating effector T cells or natural Treg cells [180–185], making it a potential treatment for GIST. In GIST cell lines, KIT exon 11 codon 557–558 deletion enhances the expression of C-X-C chemokine receptor 4 (CXCR4) [186]. Tumor cells harboring high expression of CXCR4 maybe attracted by CXCL12 secreted by hepatic cells, which partially explains the high prevalence of liver metastases of advanced GIST [186], and provides a new therapeutic target for GIST management.

Several immune-related gene sets have been constructed to evaluate the immune characteristics of GIST. Pantaleo et al. constructed the expanded IFNγ-induced immune signature (EIIS) [102] and T-cell-inflamed signature (TIS) in GIST [102], both of which are related to clinical benefit of ICIs treatment and considered as predictors of immunotherapy [187, 188]. EIIS is present in all GIST samples while TIS is highly expressed in GISTs, and they both are positively correlated with PD-L1 expression [102], suggesting that GIST may benefit from immunotherapy alone or in combination with TKIs [102]. Based on RNA-seq data, Petitprez et al. recently investigated the tumor microenvironment of 608 soft tissue sarcomas (STS) which includes 60 GISTs [189]. 25% of GIST patients belonged to sarcoma immune classes-E (SIC-E) group and such proportion was higher in GIST than in other sarcomas [189]. SIC-E subtype is characterized by strong expression of genes specific to cytotoxic lymphocytes, immune checkpoint molecules, the presence of TLSs, and may exhibit better response to PD1 blockade and longer survival [189]. In addition, Yi et al. established a prognostic model based on immunoscore [118, 119] and proved that the immunoscore is an independent prognostic factor for GIST [118, 119].

## Immunological effects of imatinib in GIST

Imatinib inhibits the proliferation and survival of GIST tumor cells primarily through suppressing KIT signaling pathway. However, some GISTs without hallmark mutations of imatinib sensitivity also show long-term responses to imatinib [136, 175], implying that the off-target effects also mediate, at least in part, the therapeutic efficacy of imatinib. For example, a case report demonstrated that a GIST patient with low KIT expression had response to imatinib treatment [190], and 6 patients without typical target mutations of imatinib were still sensitive to imatinib therapy [175]. Imatinib could kill the tumors established from several imatinib-resistant cell lines in immunocompetent mice in vivo [175], and these effects were found to be mediated mainly by the NK cells infiltrated into the tumor [136, 175]. In addition, CD8^+^ T cells were also reported to mediate part of the off-target effects of imatinib [95]. Taken together, the success of imatinib in clinic should be partially credited to its impact on the innate and adaptive immunity by modifying the tumor immune microenvironment.

**Fig. 3:**
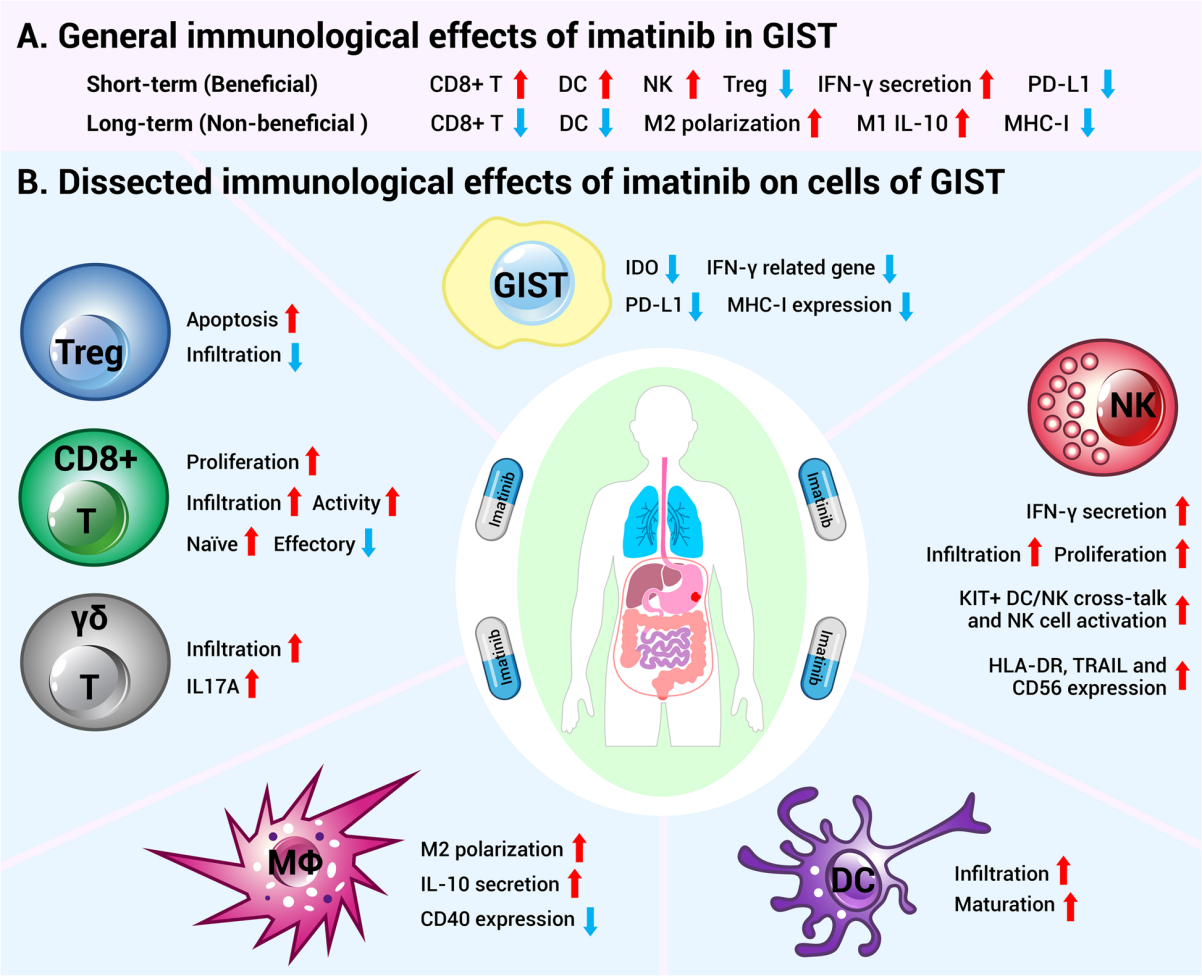
The immunomodulatory effects of imatinib in GIST. **A** The general immunological effects of imatinib in GIST. Short-term administration of imatinib enhanced the antitumor immune response via increasing the infiltration and activity of immune cells and the secretion of IFNγ. While, long-term usage of imatinib may weaken the antitumor immune response by enriching the M2 macrophages and decreasing the amounts of CD8^+^ T and DC cells, as well as the expression of MHC-I molecules. **B** Dissected immunological effects of imatinib on various types of cells within the GIST, including GIST cells, CD8^+^ T cells, γδ T cells, Treg cells, NK cells, Macrophages and DC cells. GIST: gastrointestinal stromal tumor; DC: dendritic cell; NK: natural killer cell; IFNγ: interferon gamma; PD-L1: programmed death-ligand 1; MHC-I: major histocompatibility complex class I; IDO: indoleamine 2,3-dioxygenase; Treg: regulatory T cells; MΦ: macrophages; γδ T cell: gamma delta T cell with T cell receptors (TCRs) composed of γ- and δ-chains

In general, the effects of imatinib on immune microenvironment of GIST are complex (Fig. 3A). On the one hand, imatinib brings meaningful immunologic benefit to GIST patients, such as augmentations of the infiltration and activity of CD8^+^ T cells, DC cells and NK cells, increases of IFNγ secretion, reductions in Treg cells infiltration [35, 95, 99, 113, 134, 136, 141, 169, 175, 191–194] and PD-L1 expression [35, 102], and thereby enhancing the antitumor immune responses. On the other hand, imatinib also induces immunosuppressive microenvironment. For instance, imatinib drives intratumoral macrophage M2 polarization [116], induces M1 macrophages to secrete anti-inflammatory cytokine IL-10 [97] and lowers MHC-I expression [99, 195]. Moreover, chronic imatinib therapy decreases the number of intratumoral CD8^+^ T cells and DC cells [191], and thus weakens the antitumor immune responses.

### The effect of imatinib on T cells

T cells play an important role in mediating the antitumor effect of imatinib. When compared with untreated and imatinib-resistant GIST patients, imatinib-sensitive GIST patients have more CD3^+^ and CD8^+^ T cells [95] and less Treg cells infiltration [95, 99]. As presented in Fig. 3A, imatinib treatment significantly reduced the infiltration of Treg cells [99] and imatinib exerted its antitumor effect through enhancing the function of CD8^+^ T cells [35]. In addition to studies on human GIST, there are at least four research works have examined the effects of imatinib on T cell infiltration and function in the *Kit*^*V558Δ/*+^ mouse GIST model. *First*, Balachandran et al. reported that imatinib altered the immune microenvironment of GIST by inhibiting the expression of IDO [95]. On the one hand, imatinib enhanced the infiltration and proliferation of CD8^+^ T cells in tumors, and thus augmented their antitumor immune responses which was diminished by CD8^+^ lymphodepletion [95], suggesting that the antitumor role of imatinib depends on CD8^+^ T lymphocytes. On the other hand, imatinib induced the apoptosis of Treg cells, leading to a lower infiltration level of Treg cells and higher CD8^+^ T/Treg cell ratio [95]. *Second*, Medina et al. showed that the antitumor effect of imatinib in GIST was partially mediated by DCs and effector CD8^+^ T cells [191]. Acute treatment of imatinib (1 week) increased the amount of DCs and effector CD8^+^ T cells in tumors as well as promoted the maturation of DCs. While chronic imatinib therapy decreased the infiltration of DCs and effector CD8^+^ T cells in tumors [191]. *Third*, Tieniber et al. demonstrated that imatinib reduced the infiltration of effector CD8+ T cells and increased that of naive T cells (Tn) in GIST, which were accompanied by alterations of chemokines secretion, CD8^+^ T cells recruitment and PI3K signaling within CD8^+^ T cells [196]. Interestingly, their findings about the effects of imatinib on immune cells infiltration from mouse GIST models were further validated in human GIST samples [196, 197]. *Fourth*, Etherington et al. reported that imatinib increased the γδ T cells count in GIST, and upregulated the secretion of IL17A through elevating the expression of RAR related orphan receptor C (RORC) [115]. In addition, the combined administration of imatinib and peginterferon α-2b (PegIFNα2b) induced the generation of IFNγ-producing CD8^+^ T cells [193], and the efficacy of imatinib could be enhanced by Treg cells suppression [141, 192].

### The effect of imatinib on NK cells

The therapeutic effect of imatinib in GIST can also be achieved by activating NK cells through inhibiting the KIT signaling in DCs and thus promoting the cross-talk between DCs and NK cells, resulting in the secretion of IFNγ [113, 136, 175, 192, 194]. Imatinib increases the infiltration of NK cells in GIST [99] and augments their ability to secrete IFNγ [95, 113, 134, 175, 192, 193] which was positively correlated with PFS [134, 136] and was an independent predictor of long-term survival of GIST patients with imatinib therapy [136, 175]. In addition, imatinib can activate NK cells via decreasing the expression of IDO in GIST [95]. In clinical practice, imatinib combined with IL-2 stimulates circulating NK cells in GIST patients [135, 198] and increases the expression levels of HLA-DR, TNF-related apoptosis-inducing ligand (TRAIL) and CD56 in NK cells; the abundance of HLA-DR^+^ NK cells is reported to be associated with PFS and OS in GIST [135, 198].

### The effect of imatinib on macrophages

The effect of imatinib on the immune response to GIST is not always beneficial. In GIST patients [97, 116] and *Kit*^*V558Δ/*+^ mouse GIST model [116], imatinib, through activating CCAT enhancer binding protein β (C/EBPβ) [116], drives intratumoral macrophage M2 polarization and contributes to the development of immunosuppressive microenvironment, which might partially explain the survival of tumor cells during imatinib therapy. Imatinib also downregulates the expression level of MHC-II molecules in macrophages of GIST mice [116], which contributes to the tumor progression. In addition, it has been reported that imatinib lowered the CD40 expression in macrophages and GIST cells [199], decreased the binding of CD40 to its ligands CD40L and CD154, which were expressed on activated T-helper cells, and thus led to less CD8^+^ T cell activation [199]. In vitro study also showed that imatinib increased the secretion of IL-10, an anti-inflammatory cytokine, from M1 macrophages [97] and the generation of Treg cells [97]. Therefore, modulation of the polarization status of TAM may be a promising approach for the treatment of GIST in future [97].

### The effect of imatinib on immune checkpoint and MHC molecules expression

In human GIST cell lines and *Kit*^*V558Δ/*+^ mouse GIST model, imatinib downregulates the expressions of IFNγ-related genes and IFNγ-induced PD-L1 expression by inhibiting STAT1 signaling pathway [35, 102], thereby reducing immune escape and enhancing antitumor immune response. However, imatinib also increased the expression of PD-L1 in T cells within the tumor [35]. Imatinib, through inhibiting the transcription factor ETV4, reduced the IDO expression, leading to the activation of CD8^+^ T cells and apoptosis of Treg cells [95]. Simultaneous inhibition of KIT, IDO and PD1/PD-L1 in mice was able to enhance the antitumor effect of imatinib by augmenting the function of effector T cells [35].

The expression of MHC-I molecules in GIST is highly heterogeneous, which was further decreased by the weakened type I interferons (IFNs) signaling mediated by imatinib treatment [99, 195]. In patients receiving imatinib treatment, up to 30% of GISTs completely lose and about 40% of GISTs displayed localized loss of MHC-I expression [99]. Moreover, in *Kit*^*V558Δ/*+^ GIST mice, Liu et al. reported that imatinib reduced the expression of MHC-I molecules by inhibiting type I IFNs production and signaling, attenuated tumor immunogenicity, decreased the infiltration of CD8^+^ T cells, and thus weakening the antitumor immune responses [195], which may partially explain the limited efficacy of immunotherapy for GIST patients having received prior imatinib therapy. Considering the role of type I IFNs played in affecting MHC-I expression and thus the antitumor immune responses, type I IFNs signaling has been widely exploited to improve the antitumor effect of imatinib [195]. IFNα is one of the members of type I IFNs family; in human GIST cell lines, IFNα alone is able to induce MHC-I expression, and such effect could be attenuated by imatinib [195]. In addition, the small-molecule agonist of type I IFNs has been shown to activate cGAS-STING (cyclic GMP-AMP synthase-stimulator of interferon genes) pathway and partially overcome the immunosuppressive effect caused by KIT signaling blockade, therefore enhancing the therapeutic efficacy of imatinib [195].

## Immunotherapy

**Fig. 4:**
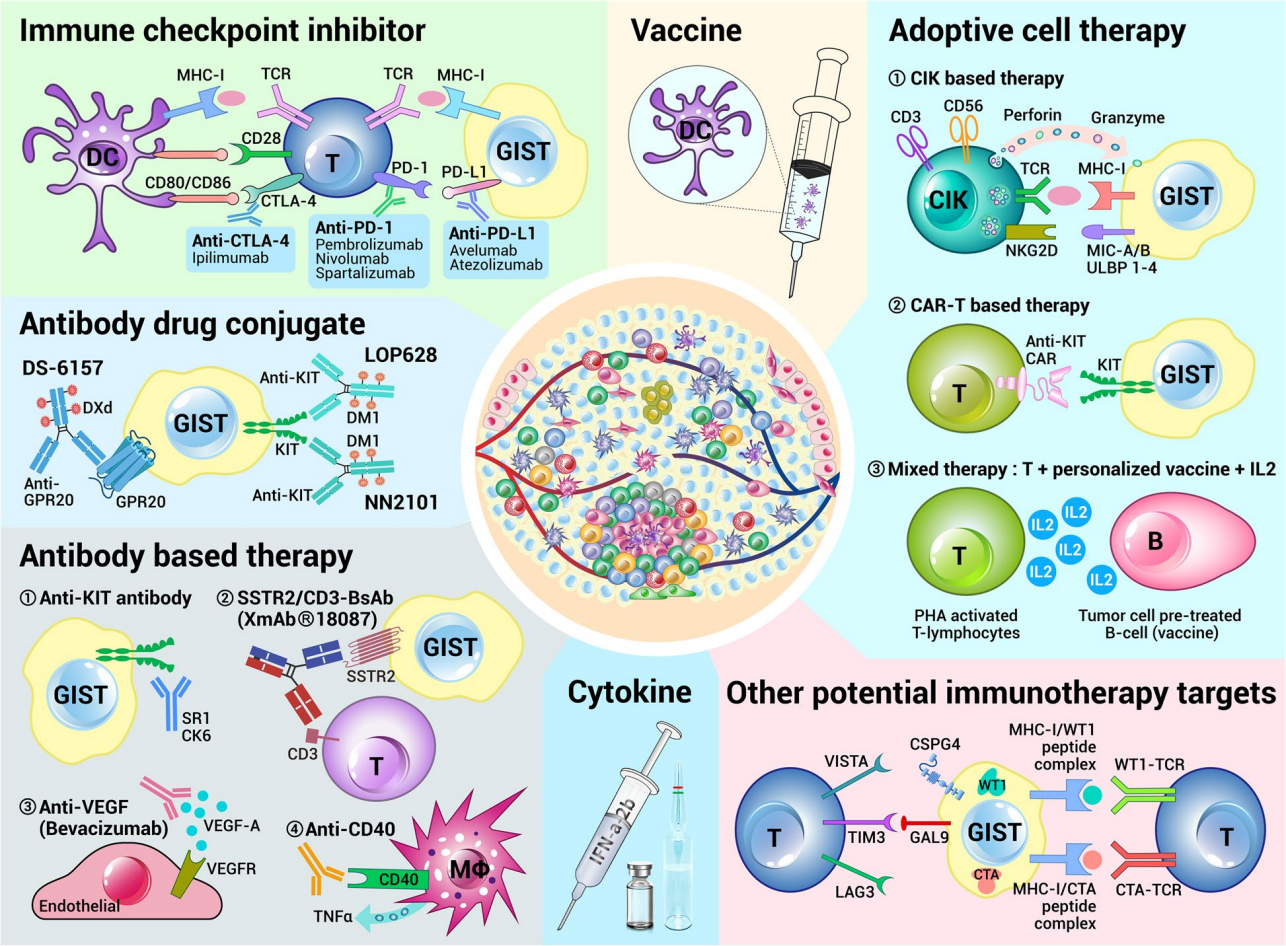
Immunotherapy strategies reported for GIST in literatures. The mostly studied immunotherapies for GIST are immune checkpoint inhibitors and cytokine therapies. Antibodies, antibody–drug conjugates, vaccines and adoptive cell therapies have also been widely evaluated in clinical or preclinical experiments in GIST. New emerging immunotherapy targets, such as WT1, CTA, CSPG4, LAG3, VISTA, Gal-9 and Tim-3, may also be exploited to develop antibody drugs or cell products to treat GIST in future. GIST: gastrointestinal stromal tumor; DC: dendritic cell; MHC-I: major histocompatibility complex class I; TCR: T cell receptors; CTLA-4: cytotoxic T-lymphocyte antigen 4; PD1: programmed cell death protein 1; PD-L1: programmed death-ligand 1; CIK: cytokine-induced killer cell; NKG2D: natural killer group 2 member D; MICA/B: MHC class I chain-related protein A and B; CAR-T: chimeric antigen receptor T; PHA: phytohemagglutinin; GPR20: G protein-coupled receptor 20; SSTR2: somatostatin receptor 2; VEGF: vascular endothelial growth factor; MΦ: macrophages; VISTA: V-type immunoglobulin (Ig) domain-containing suppressor of T-cell activation; Tim-3: T cell immunoglobulin and mucin-domain containing-3; LAG3: lymphocyte activation gene-3; CSPG4: chondroitin sulfate proteoglycan 4; Gal-9: Galectin-9; WT1: Wilms tumor protein 1; CTA: cancer testis antigen

Tumor immunotherapy, which harnesses the immune system of the host to eliminate viable tumor cells, has developed rapidly in recent years and been considered as a promising approach for cancer therapy. Since there are large amounts of immune cells infiltrated in GIST and the antitumor effect of imatinib is partly dependent on the immune system, immune cells and molecules are believed to play important roles in the occurrence and progression of GIST. Therefore, therapies by targeting the immune microenvironment of GIST have been proposed to be exploited to reactivate the antitumor immunity of the host immune system and enhance the therapeutic efficacy of imatinib, which may become new strategies to solve the bottleneck of GIST management in future. So far, several immunotherapy approaches have been reported, which include cytokine therapy, ICIs, antibody treatment, antibody–drug conjugates (ADCs), vaccine therapy and adoptive cell therapy (ACT) (Fig. 4). All clinical trials associated with the immunotherapy of GIST are registered on https://clinicaltrials.gov/ and listed in Table 2, and selected results are detailed in the following parts of this review. It is undeniable that the exploration of GIST immunotherapy is still at an early stage with controversial and even unsatisfactory findings, which may due to the immunotherapy reagents used or the patients recruited, and more research studies on GIST immunotherapy are urgently required.

**Table 2:** GIST immunotherapy clinical trials registered on https://clinicaltrials.gov/

Year	Trial ID	Phase	Immunotherapy targets	Immunotherapy agents	Combined other agents	GIST patient inclusion criteria	Status	Ref
2003	NCT00069940	I	Telomerase	Peptide vaccine	Sargramostim	Stage III or IV	Completed	[200]
2006	NCT00324987	III	VEGF	Bevacizumab	Imatinib	Metastatic/unresectable	Terminated	[201]
2008	NCT00623831	I	NA	Bacterial vaccine	NA	Metastatic, failure of imatinib and sunitinib	Completed	[202]
2008	NCT00585221	II	IFNα	PegIFNα2b	Imatinib	Metastatic/recurrent GIST patients	Terminated	[203]
2011	NCT01316263	II	PDGFRA	Olaratumab	NA	Metastatic/unresectable, failure of imatinib/sunitinib	Terminated	[204]
2012	NCT01738139	I	CTLA-4	Ipilimumab	Imatinib	Metastatic/unresectable	Recruiting	[205]
2012	NCT01643278	I	CTLA-4	Ipilimumab	Dasatinib	Metastatic/unresectable, failure of imatinib/sunitinib	Completed	[206]
2015	NCT02452424	I/II	PD1	Pembrolizumab	PLX3397	Metastatic/recurrent, failure of standard treatment	Terminated	[207]
2015	NCT02406781	II	PD1	Pembrolizumab (MK3475)	Metronomic CP	Refractory to first line imatinib and second line sunitinib	Active, not recruiting	[208]
2015	NCT02636725	II	PD1	Pembrolizumab	Axitinib	Refractory to at least first-line targeted therapy	Active, not recruiting	[209]
2015	NCT02500797	II	CTLA-4PD1	Ipilimumab Nivolumab	NA	Locally advanced/unresectable or metastatic	Active, not recruiting	[210]
2016	NCT02686944	I	NA	Intuvax (ilixadencel)	NA	Metastatic/unresectable, progressed on second, third or fourth line TKI treatment	Completed	[211]
2016	NCT02880020	II	CTLA-4PD1	Ipilimumab Nivolumab	NA	Metastatic/unresectable, refractory to imatinib	Completed	[212]
2016	NCT02834013	II	CTLA-4PD1	Ipilimumab Nivolumab	NA	Progression on standard systemic therapy, no other approved/standard therapy available	Recruiting	[213]
2017	NCT03123432	-	NA	Immunomodulating nutrients	NA	Histologically proven GIST	Completed	[214]
2017	NCT03291054	II	PD1	Pembrolizumab	Epacadostat	Unresectable/metastatic, refractory to imatinib or at least one another TKIs	Completed	[215]
2018	NCT03475953	I/II	PD-L1	Avelumab	Regorafenib	Histologically confirmed by central review	Recruiting	[216]
2018	NCT03411915	I	SSTR2, CD3	Tidutamab (XmAb18087)	NA	Advanced/metastatic/unresectable, refractory to all FDA-approved therapies	Completed	[217]
2018	NCT03609424	I/II	PD1	Spartalizumab (PDR001)	Imatinib	Metastatic/unresectable	Recruiting	[218]
2019	NCT04000529	I	PD1	Spartalizumab	TNO155, Ribociclib	Advanced, progression on or intolerance to all standard-of-care therapy per local guidelines	Recruiting	[219]
2020	NCT04276415	I	GPR20	DS-6157a	NA	Metastatic/unresectable, refractory to imatinib	Completed	[220]
2020	NCT04258956	II	PD-L1	Avelumab	Axitinib	Metastatic/unresectable, failed to standard therapy	Recruiting	[221]
2021	NCT04714983	I	NA	DNX-2440	NA	Resectable multifocal liver metastases	Recruiting	[222]
2021	NCT05152472	II	PD-L1	Atezolizumab	Imatinib	Locally advanced or metastatic, failed to at least imatinib, sunitinib and then regorafenib	Recruiting	[223]

Notes and *Abbreviations*: *“NA”* Not Applicable, *GIST* Gastrointestinal stromal tumor, *VEGF* Vascular endothelial growth factor, *IFNα* Interferon-alpha, *PegIFNα2b* Peginterferon α-2b, *PDGFRA* Platelet-derived growth factor receptor alpha, *CTLA-4* Cytotoxic T-lymphocyte antigen 4, *PD1* Programmed cell death protein 1, *CP* Cyclophosphamide, *TKIs* Tyrosine kinase inhibitors, *PD-L1* Programmed death-ligand 1, *SSTR2* Somatostatin receptor 2, *GPR20* G protein-coupled receptor 20

### Cytokine-based immunotherapy

Type I IFNs, which contain IFNα and IFNβ, are predominantly produced by macrophages, DC cells and neutrophils infiltrated in the tumor microenvironment [224]. Peginterferon α-2b (PegIFNα2b), a long-acting IFN formed by the combination of polyethylene glycol (PEG) and recombinant IFN-α-2b, has already been tested to treat GIST. Chen et al. administered 8 stage III/IV GIST patients with PegIFNα2b, and found that 4-week combination therapy induced a large amount of IFNγ and increased the infiltration of IFNγ producing CD4^+^ T, CD8^+^ T and NK cells [193, 225, 226]. After a median follow-up study of 3.6 years, they found that the overall response rate reached to 100%, and the combination therapy is superior to imatinib treatment alone [193]. To further unravel the underlying mechanisms, Zhang et al. treated the imatinib-resistant GIST cell lines with PegIFNα2b, and showed that the combination of PegIFNα2b and imatinib, but not the PegIFNα2b alone, significantly inhibited cell proliferation and induced cell apoptosis by downregulating p-mTOR (phosphorylated mammalian target of rapamycin) and BCL-2 (B-cell lymphoma-2) [227], suggesting that the combination therapy has synergistic and imatinib-resistance reversing effects [227]. Another research study reported by Pautier et al. showed that the efficacy of the combination of imatinib and IL-2 was better in GIST than renal cell carcinoma (RCC), based on 1 GIST patient [198].

### Immune checkpoint inhibitors

Immune checkpoint inhibitors (ICIs) are the most common, effective and promising immunotherapy drugs, which include anti-PD1 antibodies (pembrolizumab, nivolumab and spartalizumab), anti-CTLA-4 antibody (ipilimumab) and anti-PD-L1 antibodies (avelumab and atezolizumab). Even though ICIs have not been approved for the treatment of GIST, many clinical trials are now ongoing to explore the efficacy of ICIs in GIST patients progressing at least to imatinib (Table 3). Unfortunately, most of those clinical trials, to a large extent, were unsuccessful; almost no clear synergy was found between TKIs and ICIs. However, we cannot conclude that ICIs are ineffective, since a small number of patients with advanced GIST have achieved stable disease (SD) or partial response (PR) from ICIs and combination therapies. There is no conclusive biomarker available for now to predict and select GIST patients who may benefit from ICI immunotherapy. Several points should be taken into account to improve the efficacy of ICIs in GIST in future. *First*, according to published results which will be detailed later, GIST patients with PDGFRA D842V mutation, KIT-WT genotype, TLS presence or high PD-L1 expression seemed to have more probabilities to benefit from ICIs and should be selected preferentially [153, 189]. *Second*, nearly all clinical trials with ICIs in GIST were conducted in patients with advanced disease, whose antitumor immunity maybe weakened or heavily suppressed by long-term imatinib and multiple lines of TKI therapy, and front-line use of ICIs should be welcomed and explored. *Third*, similar to other solid tumors, more reliable biomarkers should be developed to identify patients who are sensitive to ICIs and combination therapies to achieve precision immunotherapy.

**Table 3:** The therapeutic efficacy of ICIs in GIST in published literatures

Year	Country	Phase	ICIs	Other drugs	Cases	GIST Inclusion Criteria	Key findings	Ref
2014	USA	Ib	Ipilimumab	Dasatinib	8	Failure of imatinib and sunitinib	3 GISTs achieved durable response per Choi criteria	[228]
2017	USA	Ib	Ipilimumab	Dasatinib	20	Advanced/unresectable GIST, failed to imatinib and sunitinib	13 GISTs were evaluable. 7 PR, 3 SD and 3 PD were found per Choi criteria. Synergy was not observed	[229]
2017	USA	I	Ipilimumab	Imatinib	12	Metastatic or unresectable GIST, refractory to standard therapies	1 WT gastric GIST had PR. 9 heavily pretreated GIST had no response. Synergy was not observed	[230]
2017	USA	I	Multiple	NA	9	Metastatic or unresectable advanced GIST	3 GIST patients showed SD (33%), while 1 patient showed hyper-progression after receiving ICIs	[231]
2017	France	II	Pembrolizumab	Metronomic CP	NA	Advanced GIST	The 6-month non-progression rate was 11.1%. PD1 inhibition had limited activity in advanced GIST	[232, 233]
2018	France	II	Pembrolizumab	Metronomic CP	10	Advanced GIST	The 6-month non-progression rate was 11.1%. PD1 inhibition had limited efficacy in advanced GIST, due to infiltrated macrophage and activated IDO1	[34]
2019	USA	II	Nivolumab Ipilimumab	NA	29	Advanced/metastatic GISTs refractory to at least imatinib	For nivolumab monotherapy, 7/15 GIST had SD; For combination therapy, 1/12 had PR, 2/12 had SD	[234]
2020	USA	NA	Nivolumab	NA	1	A metastatic WT GIST refractory to multiple TKIs	1 WT GIST patient showed durable response to nivolumab, progressed after 33.5 months	[235]
2020	USA	II	Nivolumab Ipilimumab	NA	18	GISTs refractory to ≥ 1 regimen(s)	Both nivolumab monotherapy and combination therapy had 9 patients, but all showed no response	[236]
2022	USA	II	Nivolumab Ipilimumab	NA	35	Advanced/metastatic GISTs refractory to at least imatinib	For nivolumab monotherapy, 10/19 had SD, For combination therapy, 1/16 had CR and 4/16 had SD	[237]
2022	India		Pembrolizumab	NA	2	Advanced/metastatic GIST	No response to pembrolizumab monotherapy	[238]

*Abbreviations*: *GIST* Gastrointestinal stromal tumor, *ICIs* Immune checkpoint inhibitors, *CP* Cyclophosphamide, *WT* wild type, *PD1* Programmed cell death protein 1, *CR* Complete response, *PR* Partial response, *PD* Progressive disease, *SD* Stable disease, *NA* Not applicable, *IDO* Indoleamine 2,3-dioxygenase

#### PD1/PD-L1 inhibitors

As mentioned above, PD-L1 and PD1 are expressed on GIST cells and infiltrating T cells, respectively, and PD1/PD-L1 inhibitors may enhance the cytotoxicity of CD8^+^ T cells against GIST cells and thus improving patients’ prognosis. In in vitro co-culture experiments of GIST cells and CD8^+^ T cells, PD-L1 blockade activates CD8^+^ T cells, and inhibits the proliferation of GIST cells and promotes their apoptosis [153]. In *Kit*^*V558Δ/*+^ mouse GIST model, anti-PD1 antibody or anti-PD-L1 antibody alone has no effect on GIST, but it can enhance the antitumor effect of imatinib through increasing the effector function of CD8^+^ T cells [35]. On the one hand, imatinib activates CD8^+^ T cells via inhibition of KIT signaling and reduction of IDO expression; on the other hand, PD1/PD-L1 blockade improves the killing activity of CD8^+^ T cells against GIST cells [35]. In clinic, a case report has shown durable responses following nivolumab treatment in a highly refractory metastatic KIT/PDGFRA-WT GIST patient [235]. Several clinical studies have investigated the therapeutic efficacy of PD1/PD-L1 inhibitors or their combinations with chemicals or antibodies in GIST [34, 238–241], but the results are unsatisfactory: most patients did not respond to these therapies and quite a few patients achieved PR or SD. In a phase 2 clinical trial, Toulmonde et al. reported that the 6-month non-progression rate of GIST patients treated with cyclophosphamide and pembrolizumab was only 11.1% [34, 232, 233]. Jiang et al. demonstrated that one GIST patient with pembrolizumab treatment achieved SD [239]. Kozak et al. evaluated the efficacy of the combination of avelumab and axitinib for unresectable/metastatic GIST but did not show their findings [240]. Curigliano et al. reported one GIST patient with combined treatment of anti-Tim-3 antibody and spartalizumab, showed progressive disease (PD) [241]. No effect was observed in two GIST patients with multisite metastases receiving pembrolizumab treatment in a retrospective study [238].

#### CTLA-4 inhibitors

CTLA-4, also known as CD152, is a transmembrane receptor on T cells. T cells lose their cytotoxicity when CTLA-4 was bound by its ligand B7, and CTLA-4 was recognized as a negative regulator of the antitumor immune response. In *Kit*^*V558Δ/*+^ mouse GIST model, the combination of imatinib and CTLA-4 inhibitors enhanced the infiltration of CD8^+^ T cells evidently, strengthened the production of IFNγ markedly, and reduced the tumor size significantly [95]. What’s more, the therapeutic efficacy of the combination therapy group was superior to that of imatinib or CTLA-4 inhibitor alone group, suggesting that synergistic effects exist of these two drugs [95]. These exciting results encouraged researchers to evaluate the effect of CTLA-4 inhibitors in GIST. A phase Ib clinical trial has evaluated the efficacy of the combination of ipilimumab and dasatinib, a second-generation TKI, in 20 advanced refractory GIST patients [229], and showed that durable Choi responses were few and synergistic effect was not observed [229]. Another phase Ib clinical trial has evaluated the efficacy of the combination of imatinib and ipilimumab in 12 metastatic/unresectable GIST patients [230], and found that only 1 gastric GIST patient with KIT/PDGFRA-WT genotype benefited from such combination therapy whom showed PR [230]. The above two clinical trials indicate that ipilimumab is clinically safe, but it seems to fail to trigger strong antitumor immune responses in patients with advanced GIST.

#### Dual inhibition of PD1/PD-L1 and CTLA-4

Two phase II clinical trials have examined the efficacy of nivolumab with or without ipilimumab in advanced GIST population, but contrasting results were obtained [236, 237]. One of the clinical trials reported by Singh et al. showed that 10 out of 19 patients receiving nivolumab alone had SD; and among the 16 patients receiving the combined therapy of nivolumab and ipilimumab, 1 patient had CR and 4 patients had SD [237]. These results suggested that immunotherapy is safe and GIST patients can benefit from ICIs. The GIST patient who had CR had primary mutation on exon 11 and secondary mutation on exon 17 of KIT gene [237]. Moreover, among the GIST patients who have benefited from ICIs for more than 6 months, 5 had tumors originated from the small intestine [237], indicating that intestinal GIST patients may be more likely to benefit from ICIs. Nevertheless, the cohort study of Alliance A091401 showed objective responses in neither 9 GIST patients undergoing nivolumab alone nor another 9 GIST patients receiving the combined therapy [236]; the median PFS of patients in single-agent nivolumab group and combined treatment group were 1.5 and 2.9 months, respectively [236]. Besides, A phase I clinical trial, with 9 metastatic or unresectable advanced GIST patients receiving different ICIs (not specified), reported that 3 patients had SD [231] and 1 patient showed hyper-progressive disease [231].

### Antibody-based immunotherapy

Monoclonal antibodies have been widely used for treating various types of tumors. Many research studies have evaluated the antitumor effects of antibodies against KIT [242–244] and CD40 [199] in mouse GIST models, and antibodies against PDGFRA [245], VEGF [246] and XmAb18087, an bispecific antibody targeting SSTR2 and CD3 simultaneously [217], in GIST patients. The findings obtained from the above studies will be systematically discussed below.

A series of research studies by Edris et al. [242, 243] showed that the anti-KIT monoclonal antibody, SR1, reduced the expression of KIT in tumor cells, strengthened the phagocytotic effect of macrophages and induced tumor cell death; its killing effect was not associated with imatinib sensitivity or resistance [242, 243]. These results suggested that anti-KIT monoclonal antibody has great potential for the treatment of GIST and might circumvent TKIs resistance, the bottleneck of the management of advanced GIST. However, CK6, another anti-KIT monoclonal antibody developed by Looy et al., failed to inhibit tumor growth in patient-derived xenograft (PDX) model, and had no synergistic antitumor effect with imatinib [244]. CD40, which is mainly expressed on the surface of APCs, is a member of the tumor necrosis factor receptor superfamily (TNFRSF) and also known as TNFRSF5. In *Kit*^*V558Δ/*+^ mouse GIST model, anti-CD40 antibody activated tumor-associated macrophages (TAMs) to produce TNFα, and enhanced the antitumor activity of imatinib [199]. The effect of anti-CD40 antibody mainly depended on TAM, followed by CD8^+^ T cells, and was independent on CD4^+^ T and B cells [199].

In GIST, the expression of VEGF is associated with poor prognosis [246]. The anti-VEGF monoclonal antibody, bevacizumab, is effective in a variety of solid tumors. Blanke et al. evaluated the therapeutic effect of bevacizumab in patients with metastatic/unresectable GIST; however, due to the small number of GIST patients enrolled and the efficacy of bevacizumab was not satisfactory, the clinical trial was terminated without consolidated conclusions [246]. The efficacy of olaratumab, an anti-PDGFRA monoclonal antibody, has been examined in 31 patients with metastatic and/or unresectable GIST (but only 20 out of 31 patients were evaluable) in 2017 [245]; no CR and PR were observed and only 5 patients had SD [245]. Tidutamab, previously known as XmAb18087, is a bispecific antibody targeting somatostatin receptor 2 (SSTR2) and CD3 simultaneously, and the former is highly expressed in GIST [247]. There is an ongoing clinical trial (NCT03411915) investigating the therapeutic effect of tidutamab on patients with advanced GIST and neuroendocrine tumors [217].

### Antibody–drug conjugates

Antibody–drug conjugates (ADC) are a kind of promising drugs in immunotherapy which chemically bond monoclonal antibodies and bioactive cytotoxic drugs. So far, two anti-KIT ADCs (LOP628-DM1 and NN2101-DM1) [248–250] and one anti-GPR20 ADC (DS-6157a) [251, 252] have been developed and tested in GIST. LOP628-emtansine (DM1) is sensitive in tumor cells with high KIT level, regardless of its mutational status, suggesting that this ADC may be used in the treatment of KIT-mutant and KIT-WT GIST [248–250], but the hypersensitivity reactions (HSRs) caused by LOP628-DM1 may constrain its usage in clinic [250]. Similarly, NN2101-DM1 was also found to inhibit the tumor growth in GIST both in vivo and in vitro, and exerted its effect regardless of KIT mutations [253]. As for DS-6157a, it is a conjugate of anti-GPR20 antibody and DNA topoisomerase I inhibitor exatecan derivative (DX-8951 derivative, DXd), which demonstrated strong cell killing activity against GIST in cell lines, cell line-derived xenograft (CDX) and PDX models [251, 252], and a phase I clinical trial examining the antitumor effect of DS-6157a in GIST patients is now ongoing [220].

### Vaccine-based immunotherapy

Vaccine is a novel approach of tumor immunotherapy. As early as 2001, Shioyama et al. reported an inoperable GIST patient whose tumor size was reduced from 11 cm in diameter to 20 mm after receiving intratumoral injection of vaccine OK432 (5 KE) [254]. Ilixadencel, also known as Intuvax, is an allogeneic DC vaccine which primes antitumor immune responses after intratumoral injection. The therapeutic efficacy of ilixadencel has been evaluated in 6 unresectable or metastatic GIST patients, and 33% of patients had radiological tumor responses [255, 256], supporting the necessity of further investigations in future.

### Adoptive cell therapy

Adoptive cell therapy (ACT) refers to collecting immune cells from patients, followed by cell expansion and genetic engineering in vitro, and then transfuses the modified cells back into the patients. Up to now, three research teams have explored the efficacy of ACT in GIST. The *first* one constructed chimeric antigen receptor T (CAR-T) cells targeting KIT in 2013 [257], and then demonstrated that such cells were able to produce IFNγ in vitro, lysed the cultured GIST cells and inhibit tumor growth in CDX model [257]. The *second* one stated, in 2019, that ACT and personalized vaccines can successfully treat recurrent GISTs [258]; eight refractory recurrent GIST patients were intravenously administrated with allogenous phytohemagglutinin (PHA)-activated T cells, personalized vaccines and low dose of interleukin-2, and 5 of them showed remissions, 1 had SD and 2 had PD after a 14-month follow-up study [258], suggesting that this therapy was safe and effective, at least for some GIST patients [258]. The *third* one got cytokine-induced killer cells (CIKs) from KIT/PDGFRA-WT GIST patients in 2022, and found that patient-derived CIKs killed autologous imatinib- and sunitinib-resistant tumor cells either directly or indirectly [259].

### Other potential immunotherapy targets

In addition to the aforementioned immunotherapies, here are some other potential immunotherapy targets for GIST. Both M2 macrophages, one of the most abundant cells in the microenvironment of GIST [97, 101, 104] and Treg cells, which highly express CCR8 [97, 178], are involved in immune escape, therefore, they are considered as the targets for GIST immunotherapy. Moreover, LAG3 [35] and Tim-3 [35, 108] are present in tumor-infiltrating lymphocytes, suggesting that they may participate in immune escape of GIST and are the potential targets for immunotherapy. CTAs have been reported to be expressed in 26.7%-40% of GIST patients [168–170]; immune cells against these CTAs molecules will be established in the near future to treat GIST. In addition, Wilms tumor protein 1 (WT-1) [260] and chondroitin sulfate proteoglycan 4 (CSPG4) [261], which are overexpressed on GIST cells, are also regarded as new potential immunotherapy targets of GIST.

## Research models for GIST

**Fig. 5:**
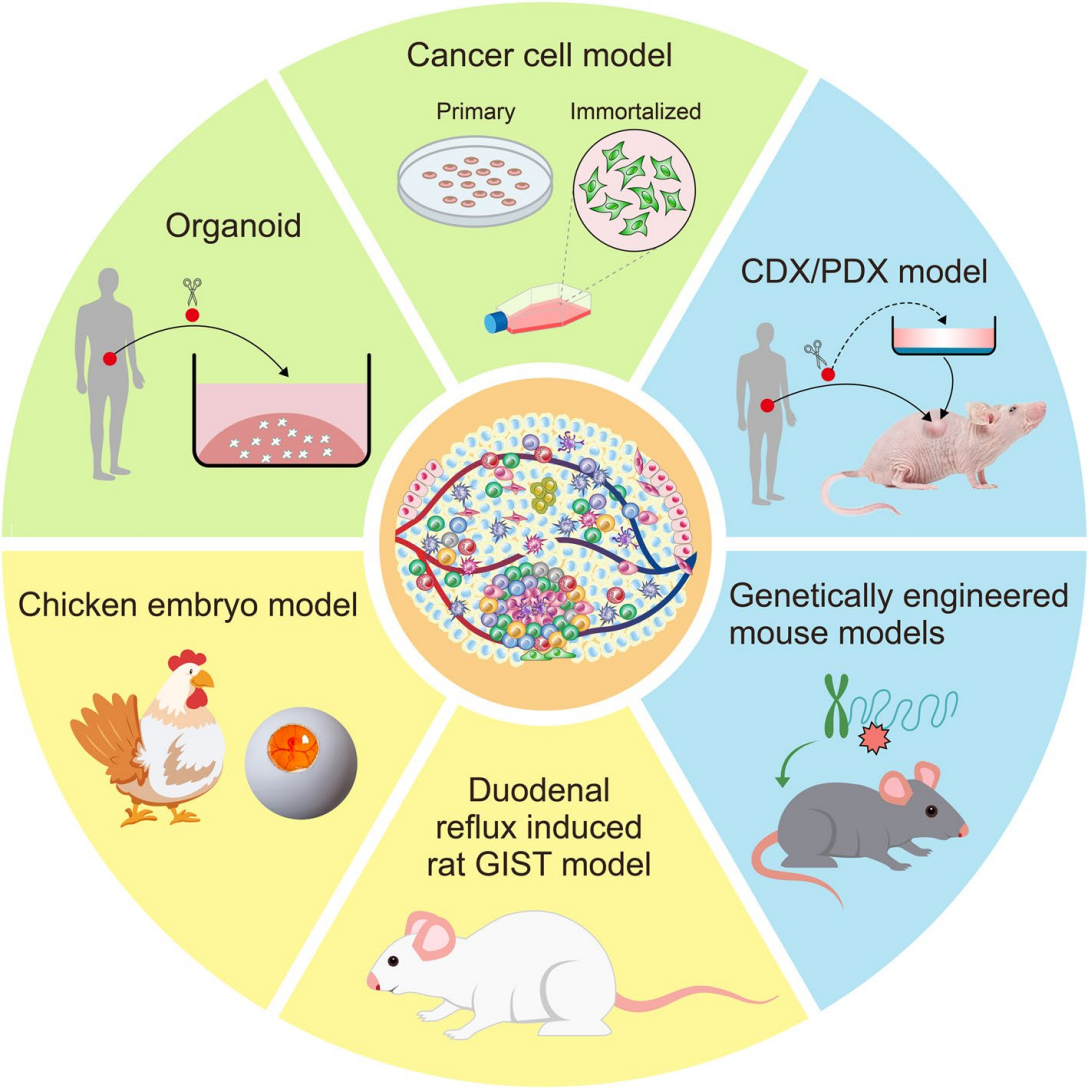
Models for GIST research in literatures. Cell lines, CDX, PDX and genetically engineered mouse models are the most frequently used models. chicken embryo model and the duodenal reflux induced rat model have been reported for GIST but haven't been widely applied. Organoid models are emerging and hold brilliant future. CDX: cell line-derived xenograft; PDX: patient-derived xenograft

Basic and translational researches of GIST cannot be accomplished without appropriate experimental models, such as cells, animals and organoids, and the available models for GIST in literatures are illustrated in Fig. 5. The cell models mainly include primary cell culture [262] and immortalized cell lines; due to convenience and cost-effectiveness, cell lines are widely used in GIST studies, for example, GIST882, GIST-T1, GIST430, GIST48 and mouse S2 cells [116, 191, 199, 263, 264]. The major characteristics of GIST cell lines are described in Table 4. The reported animal models include the rat GIST model [265], chicken embryo model [266], lymph node metastasis mice [267], peritoneal dissemination mice [268], spleen-to-liver metastasis mice [176, 177, 269, 270], PDX and genetically engineered mouse models (GEMMs), with the latter two are the most widely used animal GIST models. The PDX model, which is established by implantation of tumor tissues [251, 271, 272] or primary cells derived from GIST patients [273–275] into immunodeficient mice, keeps most of the characteristics of primary GISTs at the histopathological, biological and genetic levels, but is not suitable for immune research. The GEMMs refer to mice that are modified by genetic engineering technology and develop GIST spontaneously with competent immune system. In view of the technical difficulties in construction and high cost during maintenance, only limited GEMMs for GIST have been successfully established, which are summarized in detail in Table 5. Tumor organoids are three-dimensional structures constructed by in vitro 3D culture of tumor tissues collected from patients; they not only maintain the morphological structure of tumors, but also keep the tumor gene expression and heterogeneity. As for the organoids for GIST, they are still in an initial stage; only Cao et al. [276] and Forsythe et al. [277] reported the establishment of GIST organoids and figured out that organoids may have the potential for the precise treatment of GIST.

**Table 4:** Commonly used cell lines for GIST research in literatures

Cell line	Mutation	KIT expression	Imatinib sensitivity	Source	Ref
Ba/F3	Engineered to express c-Kit or PDGFRA mutants	NA	NA	Murine pro-B cell line	[11, 278, 279]
GIST882	KIT Ex.13: K642E missense point mutation (Homo)	Positive	Sensitive	TKI-naïve metastatic human GISTs	[280]
GIST-T1	KIT Ex.11: in-frame deletion of 57 bp (V570-Y578) (Heter)	Positive	Sensitive	Untreated metastatic plural tumor of gastric GIST	[281]
GIST544	KIT Ex.9: AY503-504ins mutation	Negative	Unclear	Short-term culture from a GIST	[282]
GIST430	KIT Ex.11: in-frame deletion (V560-L576) (Heter) KIT Ex.13: V654A missense point mutation (Heter)	Positive	Resistant	GISTs developed resistance to imatinib therapy after initial response to imatinib therapy	[283, 284]
GIST48	KIT Ex.11: V560D missense point mutation (Homo) KIT Ex.17: D820A missense point mutation (Heter)	Positive	Resistant	GISTs developed resistance to imatinib therapy after initial response to imatinib therapy	[283, 284]
GIST GDG1	Unclear	Positive	Resistant	GIST patient progressed during imatinib treatment	[285, 286]
GIST62	KIT Ex.11: in-frame deletion (MYEVQWK552-558 T) (Heter)	Negative	Resistant	Untreated KIT-positive GIST	[284]
GIST-DR	No Kit gene mutation	Positive	Sensitive	Rat GIST induced by duodenal reflux	[265]
GIST522	KIT Ex.11: in-frame deletion (EVQWK554-558) (Heter)	Negative	Resistant	Imatinib-resistant, progressing GIST	[287]
GIST-H1	Unclear	Positive	Unclear	Unclear	[288]
GIST-PSW	KIT Ex.11: K558_G565delinsR mutation	Positive	Sensitive	GIST patients radiologically progressing under imatinib	[289]
GIST-BOE	KIT Ex.9: A502_Y503dup	Positive	Resistant	GIST patients radiologically progressing under imatinib	[290]
GIST5	KIT Ex.11: mutations	Negative	Unclear	Established from imatinib- treated GISTs	[291]
GIST474	KIT Ex.11: mutations	Negative	Unclear	Established from imatinib- treated GISTs	[291]
GIST867	Unclear	Unclear	Resistant	Intestinal GISTs treated with imatinib	[292]
S2 cells	KIT Ex.11: V558Δ mutation (Heter)	Negative	Resistant	*Kit*^*V558Δ/*+^ mouse GIST tumor	[116]
GK1C	KIT Ex.11: in-frame deletion (550–558)	Positive	Sensitive	High metastatic risk GISTs	[293]
GK3C	KIT Ex.11: in-frame deletion (591–592)	Positive	Sensitive	High metastatic risk GISTs	[293]
HG129	KIT Ex.11: 45 bp insertion between F591-592G	Positive	Sensitive	Untreated primary gastric GIST	[294]
GIST226	KIT Ex.11: in-frame deletion (P551-W557) (Homo) KIT Ex.17: Y823D point mutation (Homo)	Negative	Resistant	Unclear	[295]
HG209	KIT Ex.11: in-frame deletion (YIDPTQL 570–576) KIT Ex.17: D816H point mutation	Negative	Resistant	imatinib- and sunitinib-resistant peritoneal metastasis	[296]
GIST-5R	Δ560 − 578/T670I	Positive	Resistant	Unclear	[297]
GIST54	Unclear	Negative	Resistant	Unclear	[298]

Cell lines are listed in the order of the year they were firstly reported, and only the earliest papers are referenced here. *Homo* homozygous mutation, *Heter* heterozygous mutation, *Ex* Exon

**Table 5:** GEMMs described in literatures

Animal models	Mutations and human counterparts	Key features	Ref
*Kit*^*V558Δ/*+^ Mice	Germ line deletion of V558 of Ex. 11 of mouse *Kit* gene. KIT V559 of Ex. 11 in human	Heterozygotes had cecal GIST with high penetrance, and showed diffuse ICC hyperplasia from oesophagus to large intestine	[299]
*Kit*^+*/K641E:Neo*^ mice *Kit*^*K641E:Neo/K641E:Neo*^ mice	Germ line K641E mutation at Ex. 13 of mouse *Kit* was introduced in mice. Corresponding to human KIT K642E mutation at Ex. 13	Both homozygotes and heterozygotes had cecal GIST and showed diffuse ICC hyperplasia from oesophagus to large intestine. Homozygotes died 30 weeks after birth. Heterozygotes have smaller GISTs	[300]
*Kit*^*D818Y/*+^ mice *Kit*^*D818Y/D818Y*^ mice	Germ line *Kit* D818Y mutation was introduced in mice. Corresponding to human KIT D820Y mutation at Ex. 17	Both homozygotes and heterozygotes had cecal GIST and showed diffuse ICC hyperplasia from oesophagus to large intestine. Homozygotes had larger cecal tumor	[301]
*Kit*^*V558Δ;T669I/*+^ mice	Mouse *Kit* T669I mutation corresponding to human KIT T670I mutation. A mouse model for imatinib-resistant GIST	Mutant mice had a prolonged lifespan, developed smaller cecal GIST and more pronounced ICC hyperplasia in the stomach and colon, when compared to *Kit*^*V558Δ/*+^ mice	[302]
*Ptch*^*flox/flox*^-*Lys*^*Mcre*±^ mice	Cell specific loss of expression of *Ptch* from lysozyme M-expressing cells	About 82% mutant mice had GIST-liked tumors, which sometimes expressed *Pdgfrα*, but not *Kit*, arising from the wall of stomach or intestine	[303]
*Etv1*^*flox/flox*^; *Kit*^*Δ558V/*+^; *Rosa26*^*CreERT2/CreERT2*^ mice	Inducible *Etv1* knockout mice, suitable for the study of the function of *Etv1* in GIST	Vehicle-treated mice had an identical phenotype to the *Kit*^*Δ558V/*+^ mice, with GIST-like tumors in cecum and ICC hyperplasia in large intestine and stomach	[304]
*Kit*^*V558Δ; Y567F/Y567F*^ mice	The KIT phosphorylation site was blocked by introducing Y567F mutation	Mutant mice had similar but significantly smaller cecal GIST to that of *Kit*^*V558Δ/*+^ mice, had less ICC hyperplasia in stomach and colon	[305]
*Etv1*^*CreERT2*^; *Braf *^*CA/*+^; *Trp53*^*fl/fl*^ mice	A mouse model of aggressive imatinib-resistant human BRAF^V600E^-mutant GIST	Mutant mice developed ICC hyperplasia and multifocal GIST-like tumors within the gastrointestinal tract with 100% penetrance after the tamoxifen administration	[306]
*Lrig1*^*CreERT2/*+^; *Braf*^*LSL−V600E/*+^ mice	Mutant *Braf* expression could be induced in *Lrig1* expressing cells	Activation of mutant Braf in mice resulted in oral tumors (squamous papillomas), and ICC hyperplasia in mouse stomach and colon	[307]
*Myh11*^*CreERT2*^; *Braf*^*LSL−V600E/*+^ mice	Mutant *Braf* expression could be induced in *Myh11* expressing smooth muscle cells	Approximately 36.4% mice showed overt GIST either in stomach, cecum or bladder. ICC hyperplasia was also found in mutant mice	[307]
*Lrig1*^*CreERT2/*+^; *Rosa*^*LSL−Kitc/*+^ mice	Mutant KIT expression could be induced in *Lrig1* expressing cells	Mutant mice developed small GIST-like lesions in the muscularis propria of the stomach and intestine	[307]
*Kit*^*V558Δ;V653A−NEO/*+^; *Etv1*^*Cre−ERT2/*+^ mice	Corresponding to human KIT V654A mutation, a model of imatinib resistant GIST caused by secondary mutation	More than 95% mice developed cecal GIST after tamoxifen administration. Mutant mice had larger tumors and decreased survival when compared to *Kit*^*V558Δ/*+^ mice	[308]

The immune microenvironment plays an important role in the pathogenesis and progression of GIST; however, most of the aforementioned experimental models for GIST lack in vivo immune microenvironment. Relatively speaking, the GEMMs better preserve the in vivo immune microenvironment, making them the best approach to examine the immuno-oncology of GIST and to develop drugs for GIST immunotherapy. So far, the established GEMMs mainly focus on mutations of KIT and BRAF, and mouse GIST models for other gene mutations are still underexplored. Moreover, most GIST mice driven by germline gene mutations develop tumors mainly in the cecum, which are not consistent with the conditions of human GISTs which almost originate from stomach and small intestine. Therefore, mouse GIST models that resemble human GIST conditions are urgently needed.

## Conclusions and future perspectives

In summary, GISTs are predominantly populated by macrophages and T cells, and to a lesser extent, by NK and B cells, and the distribution of other immune cell subtypes are unclear up to now. The levels of intratumoral immune cells are associated with clinical outcomes and clinicopathological features of GIST patients, while their functions and underlying molecular mechanisms in the tumorigenesis, progression, metastasis and drug resistance of GIST require more attentions. The numbers of intratumoral immune cells are related to driver gene mutations, metastases and anatomical locations, and are modulated by various lines of targeted therapies, however nearly no research work have yet answered that how these factors reshaped the immune landscape of GIST exactly. The immune profiling analyses are mainly accomplished by immunohistochemistry (IHC), and rarely by flow cytometry or immunofluorescence, therefore, available conclusions need to be further confirmed and new immunotherapy targets need to be identified by more sophisticated techniques, such as multiplex immunohistochemistry and immunofluorescence (mIHC/mIF), which simultaneously evaluate the expression of multiple biomarkers within a single tissue slice [309], and single-cell multiomics which dissect the various intratumoral immune cell subtypes and identify the key regulators of antitumor immunity at high resolutions [310].

The microenvironments of GISTs are grossly immunosuppressive, which are mediated by the infiltration of M2 macrophages [34] and Treg cells [97], high-expression of IDO on GIST cells [124] and immunosuppressive receptors on NK cells [134], and deficient expression of MHC-I on APCs [97]. Though CD8^+^ T cells are enriched in GIST [127], the CD8^+^ T/Treg cell ratio is relative low [97], and might be further inhibited by PD-L1 on GIST cells [124], and by glucocorticoid-induced TNF receptor-related protein (GITR) and inducible T-cell costimulator (ICOS) on Treg cells [166]. Besides, NK cells were found to be CD69 negative, suggesting they are in a dysfunctional state. Taken together, several factors contribute to the development of immunosuppressive microenvironments [97], which might mediate the primary resistance to ICIs in GIST [34].

Imatinib exerts antitumor activity by its direct killing ability on the tumor cells and indirect off-target effects on immune cells. The immunological effects of imatinib in GIST are complex, analytical researches are still necessary to dissect the exact roles of imatinib in GIST immuno-oncology. In general, short-term usage of imatinib enhances the host antitumor immune responses by activating CD8^+^ T cells, DC cells and NK cells, and inhibiting Treg cells. While chronic administration of imatinib may contribute to the immunosuppressive microenvironment of GIST by inducing the M2 polarization of macrophages, the loss of MHC-I expression, and the decrease of the activity of CD8^+^ T cells, and all these changes maybe the reasons of low response rates to ICIs in heavily pretreated GIST patients.

Several clinical and pre-clinical trials have been conducted in GIST to examine the effects of immunotherapies, such as cytokines, ICIs, antibodies, ADCs, vaccines and ACTs. Some of the findings are promising, while the others are controversial and unsatisfactory, especially for ICIs, which include anti-PD1/PD-L1 and anti-CTLA-4 antibodies and are the most widely studied drugs in heavily pretreated GIST patients. The relative low expression of PD1/PD-L1 and the loss of MHC-I in GIST may partially explain the low responsiveness to ICIs, which suggests that other new emerging immunotherapy targets, such as M2 macrophages, Treg cells, LAG3, Tim-3, CTAs, WT-1 and CSPG4, deserve more attentions. Since a few GIST patients did benefit from ICIs, which are consistent with other solid tumors, reliable biomarkers should be developed, patients should be carefully selected, combination therapy and front-line immunotherapy should be warmly recommended and encouraged in future investigations [311].

The establishments of GIST cell lines, CDX/PDX models and GEMMs considerably facilitate the research and understanding of the pathogenesis, progression, metastasis and drug resistance, and noticeably accelerate the screening and development of novel therapeutic targets and drugs, which in turn improve the management and outcome of GIST patients. However, these reported models cannot fully recapitulate the features of human GIST, such as the morphology, gene expression pattern and heterogeneity, and preserve the immune microenvironment, which seriously hinder the immuno-oncology research and the development of immunotherapy drugs. GEMMs harbor competent immune microenvironment but develop tumors in the cecum, which are scarcely observed in human GISTs. GIST models that more resemble the human GISTs in anatomy, genotype, phenotype and drug resistance are urgently needed. In addition, vascularized and immunized organoids [312] may be other models with brilliant prospect for studying GIST in near future.

## Fundings

This study was supported by grants from the Guangdong Provincial Key Laboratory of Digestive Cancer Research (2021B1212040006), the Guangdong-Hong Kong-Macau University Joint Laboratory of Digestive Cancer Research (2021LSYS003), the Shenzhen Key Laboratory of Chinese Medicine Active Substance Screening and Translational Research (ZDSYS20220606100801003), the Shenzhen-Hong Kong-Macau Technology Research Program (Type C) (SGDX20201103092601008), the Guangdong Basic and Applied Basic Research Foundation (2022A1515110194, 2021B1515120069, 2022A1515110156), the National Natural Science Foundation of China (U20A20379, 82220108013), the Shenzhen Fundamental Research Program (JCYJ20220530142203008, JCYJ20190809142807444), the Shenzhen Sustainable Project (KCXFZ20200201101059392), and the Sanming Project of Medicine in Shenzhen (SZSM201911010).

## Data Availability

Not applicable.

## Abbreviations

ACT: Adoptive cell therapy
ADCC: Antibody-dependent cellular cytotoxicity
ADCs: Antibody–drug conjugates
AFIP: Armed Forces Institute of Pathology
APCs: Antigen presenting cells
BCL-2: B-cell lymphoma-2
BIRC3: Baculoviral IAP repeat-containing protein 3
BRAF: B-Raf proto-oncogene
CAR-T: Chimeric antigen receptor T
CCL: C–C motif chemokine ligand
CCR: C–C chemokine receptor
CDC: Complement-dependent cytotoxicity
CDX: Cell line-derived xenograft
C/EBPβ: CCAT enhancer binding protein β
cGAS-STING: Cyclic GMP-AMP synthase-stimulator of interferon genes
CIKs: Cytokine-induced killer cells
CR: Complete response
CSPG4: Chondroitin sulfate proteoglycan 4
CTAs: Cancer testis antigens
CTLA-4: Cytotoxic T-lymphocyte antigen 4
CXCL: C-X-C motif chemokine ligand
CXCR: C-X-C chemokine receptor
DC: Dendritic cell
DFS: Disease-free survival
DKK4: Dickkopf 4
DM1: Emtansine
DXd: DX-8951 derivative
EC: Extracellular domain
EIIS: Expanded IFNγ-induced immune signature
ETV4: ETS variant transcription factor 4
FDA: Food and Drug Administration
GAGE: G antigen
Gal-9: Galectin-9
γδ T cell: Gamma delta T cells with TCRs composed of γ- and δ-chains
GEMMs: Genetically engineered mouse models
GIST: Gastrointestinal stromal tumor
GITR: Glucocorticoid-induced TNF receptor-related protein
GPR20: G protein-coupled receptor 20
HALP: The combined index of hemoglobin, albumin, lymphocyte, and platelet
HGF: Hepatocyte growth factor
HLAs: Human leukocyte antigens
HSRs: Hypersensitivity reactions
ICCs: Interstitial cells of Cajal
ICIs: Immune checkpoint inhibitors
ICOS: Inducible T cell costimulator
IDO: Indoleamine 2,3-dioxygenase
IFN: Interferon
Ig: Immunoglobulin
IHC: Immunohistochemistry
IL: Interleukin
JM: Juxtamembrane domain
LAG3: Lymphocyte activation gene-3
LMR: Lymphocyte-to-monocyte ratio
LWR: Lymphocyte-to-white cell ratio
MΦ: Macrophages;
M1: M1 macrophages that are classically activated
M2: M2 macrophages that are alternatively activated
MAGE: Melanoma-associated antigen
MDSCs: Myeloid-derived suppressor cells
MHC: Major histocompatibility complex
MICA/B: MHC class I chain-related protein A and B
mIHC/mIF: Multiplex immunohistochemistry and immunofluorescence
MLR: Monocyte-to-lymphocyte ratio
ML-CTC: Macrophage-like circulating tumor cells
MWR: Monocyte-to-white cell ratio
NAR: Neutrophil-to-albumin ratio
NCR1: Natural cytotoxicity triggering receptor 1
NF1: Neurofibromatosis type 1
NF-kB: Nuclear factor kappa-light-chain-enhancer of activated B cells
NK: Natural killer cell
NKG2D: Natural killer group 2 member D
NKp30: Natural killer cell p30-related protein
NKp46: Natural killer cell p46-related protein
NKT: Natural killer T cells
NLR: Neutrophil-to-lymphocyte ratio
NSCLC: Non-small cell lung cancer
NWR: Neutrophil-to-white blood cell ratio
NY-ESO-1: New York esophageal squamous cell carcinoma-1
OS: Overall survival
PBMC: Peripheral blood mononuclear cell
PD: Progressive disease
PD1: Programmed cell death protein 1
PDGFRA: Platelet-derived growth factor receptor alpha
PDX: Patient-derived xenograft
PD-L1: Programmed death-ligand 1
PEG: Polyethylene glycol
PegIFNα2b: Peginterferon α-2b
PFS: Progression-free survival
PHA: Phytohemagglutinin
PLR: Platelet-to-lymphocyte ratio
PNI: Prognostic nutritional index
PR: Partial response
PWR: Platelet-to-white cell ratio
p-mTOR: Phosphorylated mammalian target of rapamycin (mTOR)
RAS: Rat sarcoma viral oncogene
RCC: Renal cell carcinoma
RFS: Recurrence-free survival
RORC: RAR related orphan receptor C
ROS: Reactive oxygen species
SD: Stable disease
SDH: Succinate dehydrogenase
SIC-E: Sarcoma immune classes-E
SII: Systemic immunoinflammatory index
SPARCL1: Secreted protein acidic and rich in cysteine-like 1
SSTR2: Somatostatin receptor 2
STS: Soft tissue sarcoma
TAM: Tumor-associated macrophage
TCR: T cell receptor
TGFβ: Transforming growth factor beta
Th1: T helper type 1 cell
Th2: T helper type 2 cell
TILs: Tumor-infiltrating lymphocytes
Tim-3: T cell immunoglobulin and mucin-domain containing-3
TIS: T-cell-inflamed signature
TK: Tyrosine kinase domain
TKIs: Tyrosine kinase inhibitors
TLS: Tertiary lymphoid structures
TM: Transmembrane domain
Tn: Naive T cells
TNFα: Tumor necrosis factor alpha
TNFRSF: Tumor necrosis factor receptor superfamily
TRAF1: Tumor necrosis factor receptor (TNFR) associated factor 1
TRAIL: TNF-related apoptosis-inducing ligand
Treg: Regulatory T cell
VEGF: Vascular endothelial growth factor
VISTA: V-type immunoglobulin (Ig) domain-containing suppressor of T-cell activation
WT: Wild type
WT-1: Wilms tumor protein 1
